# Alternating High‐Fat and Polysaccharide Diets Modulates Gut Phage‐Bacterial Interplay

**DOI:** 10.1002/advs.202516916

**Published:** 2026-03-12

**Authors:** Fengxiang Zhao, Ruiqi Zhang, Rujun Wei, Huimin Fan, Yongfei Hu, Wenyu Shi, Jinfeng Wang

**Affiliations:** ^1^ College of Food Science & Nutritional Engineering China Agricultural University Beijing China; ^2^ State Key Laboratory of Animal Nutrition and Feeding College of Animal Science and Technology China Agricultural University Beijing China; ^3^ College of Biological Sciences China Agricultural University Beijing China; ^4^ Key Laboratory of Fruit and Vegetable Processing of Ministry of Agriculture and Rural Affairs & Beijing Key Laboratory for Food Non‐Thermal Processing China Agricultural University Beijing China

**Keywords:** diet, fucoidan, gene exchange, gut virome, phage‐bacteria interaction

## Abstract

Phages dominate the human gut virome and are known for their ability to prey on bacteria and shape microbiota. However, their response to diet has only been elucidated using small‐scale studies. By integrating a massive meta‐analysis of 6932 diet‐associated metagenomes with a time‐resolved mouse model of a high‐fat diet and polysaccharide intake, the impact of diet on the gut virome and phage–bacterial interactions was systematically characterized. Diet types, particularly high‐fat and polysaccharide‐rich diets, exert the strongest shaping force on the gut virome, enhancing the crosstalk between phages and bacteria. High‐fat diets promote changes in phage abundance across a broad taxonomic range, from 34.21% to 50.00%, drive phages of diet‐associated bacteria toward a lytic lifestyle, and remarkably enrich auxiliary metabolic genes related to amino acid metabolism. Conversely, fucoidan reversed HFD‐induced dysbiosis and enhanced phage‐mediated horizontal gene transfer by 8.5‐fold relative to the baseline. crAssphages and *Parabacteroides* phages may be important contributors, broadly supporting horizontal gene transfer and auxiliary metabolism or strengthening phage–host interactions in polysaccharide interventions, including fucoidan supplementation. These findings provide a comprehensive landscape of diet‐driven cross‐kingdom interactions and phage‐mediated gene exchange in the gut, offering new insights into potential strategies for precise nutritional interventions targeting the intestinal microbiota.

## Graphical abstract

This study reveals how alternating high‐fat and polysaccharide diets reshape the human gut virome and enhance phage‐bacteria interactions. Using large‐scale metagenomic meta‐analysis and a time‐resolved mouse model, the authors show that diets strongly modulate phage abundance, lifestyle, and gene exchange, offering new insights into nutrition‐guided microbiome modulation.

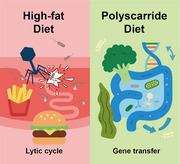

## 1 Introduction

In addition to host genetics [1], geographic environment [2], and urbanization [3], dietary interventions are particularly likely to disrupt the homeostasis of the gut microbiota [4]. As the most common and readily modifiable external factor, diet can directly promote or inhibit the growth of specific bacteria and indirectly reshape the microbiota by affecting host metabolism and immune systems [5]. Previous studies have predominantly focused on single dietary types or components and their effects on bacterial communities and host physiology, while overlooking cross‐kingdom microbial interactions and gene exchange.

Recently, the gut virome, an important component of the human microbiome, has gained increasing attention and is recognized as a key player in health regulation [6, 7, 8, 9, 10, 11]. Bacteriophages (phages), which dominate the human gut virome, not only trigger cellular immune responses [12, 13] but also dynamically modulate the structure and function of bacterial communities, thereby influencing host physiology [14, 15, 16, 17]. Phages interact with bacteria and regulate bacterial communities through various mechanisms, including lifestyle transitions, mediation of horizontal gene transfer (HGT), and dissemination of auxiliary metabolic genes (AMGs). These diverse modes of interaction create significant challenges in elucidating phage‐mediated microbial regulation and responses to external perturbations. A major knowledge gap remains regarding how phages respond to dietary changes and interact with bacteria.

Emerging evidence indicates that the gut virome, particularly phages, is shaped by diet and contributes to diet‐induced physiological changes in the host. For example, the transplantation of virus‐like particles (VLPs) from low‐fat diet mice to high‐fat diet recipients reduces bacterial overgrowth in the small intestine [18]. This intervention alters the composition of gut bacteria and viruses, affects the plasma metabolome, and modifies gene expression profiles related to obesity and type 2 diabetes (T2D), while significantly suppressing weight gain and improving glucose tolerance [19]. High‐fat, high‐sugar diets have been shown to enrich lysogenic phages [20] and enhance the pathogenicity of commensal gut bacteria [21]. In contrast, diets rich in plant polysaccharides restore microbial balance by modulating phage lifestyles [22]. While these studies highlight the potential of the virome in mediating the dietary regulation of gut microbiota and host health, comprehensive analyses are still needed to fully characterize the scope and dynamics of phage responses to diet, as well as their interactions with the bacteriome, using large‐scale datasets encompassing diverse dietary types and components.

In this study, we integrated diet‐related gut microbiome metagenomic data and designed a time‐resolved animal experiment to systematically analyze the effects of dietary intake on cross‐kingdom interactions and the dynamics between phages and bacteria in the gut virome. Our findings highlight the role of the virome, particularly bacteriophages, in mediating gut microbiota responses to dietary intake. This study revealed that alternating high‐fat and polysaccharide‐rich diets reshaped the microbiota and its metabolic functions by enhancing phage–host interactions, offering new perspectives for precision nutritional interventions.

## 2 Results

### 2.1 Distinct Dietary Types Specifically Reshape the Gut Virome

**FIGURE 1:**
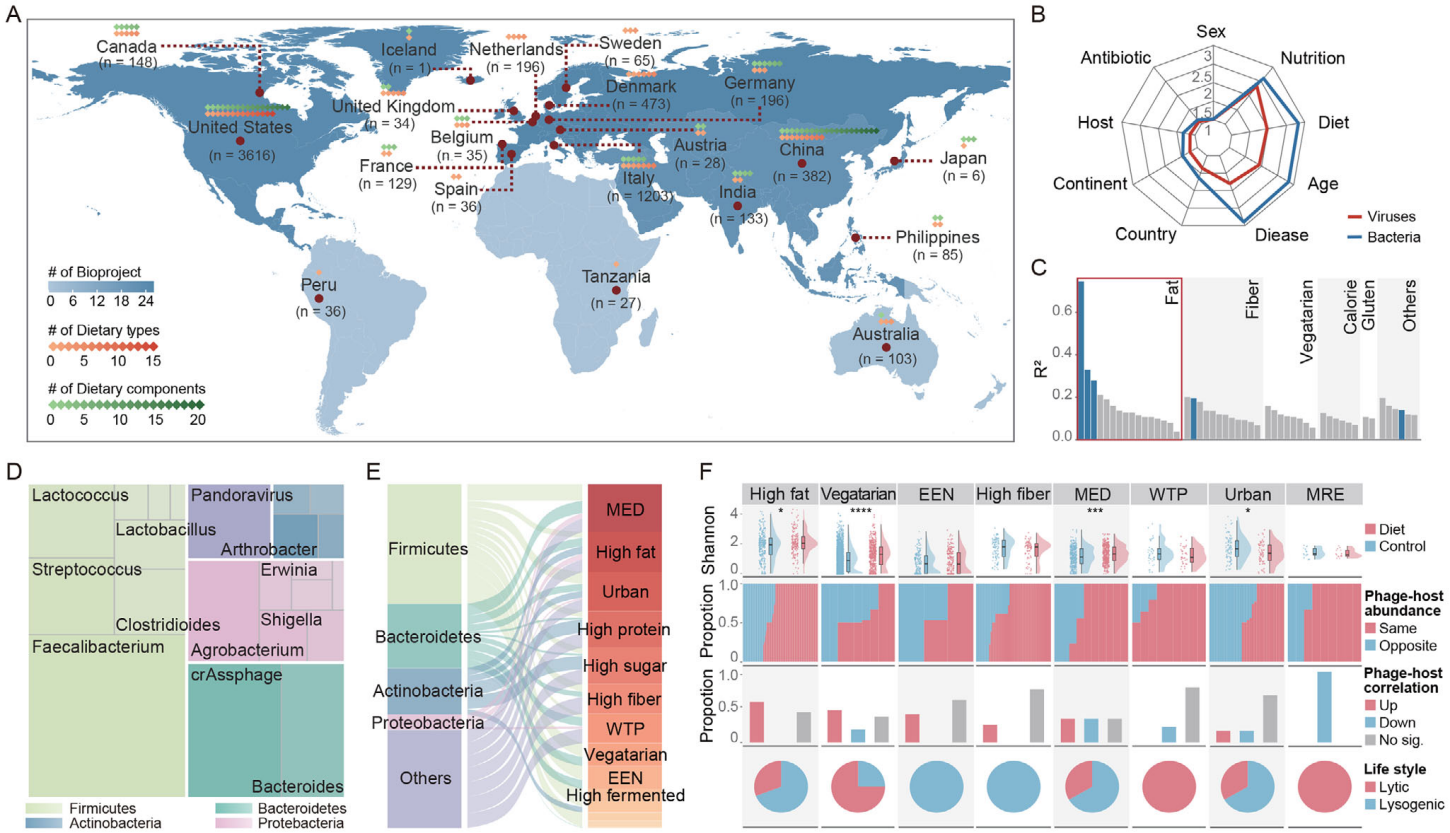
Diet‐specific remolding of the gut virome. (A) Geographic distribution of 6932 diet‐associated metagenomic samples. The global heatmap displays the number and distribution of 62 Bioprojects (retrieved from NCBI SRA) by continent. Each point represents a different country and contains information about the number of individuals sampled, diet types (red) and/or dietary components (green). (B) Diet explains more variation in the virome than in the bacteriome. This radar chart compares the effect size (PERMANOVA *R* ^2^, Bray‐Curtis dissimilarity, 999 permutations) of various factors on gut virome (red line) and bacteriome (blue line) at species level. (C) Effect size of different dietary types on the virome. Dietary variables are ranked by their explanatory power (*R* ^2^). Only those with statistical significance (PERMANOVA, *p* < 0.05) were shown in blue. (D) Gut bacteriophages in response to dietary intake. Colors correspond to phage taxa (grouped by host phylum) with significant abundance changes in response to dietary intake (determined by LEfSe, LDA score > 2, Kruskal–Wallis *p* < 0.05). The area of each segment is proportional to the percentage of responsive phages within that taxonomic group. (E) Mapping phage responses across diverse dietary types. This alluvial plot shows the response of different phage groups (classified by host phylum) to 15 specific dietary types. The width of each flow is proportional to the percentage of phages within a phylum that are significantly changed in abundance (LEfSe, LDA score > 2, Kruskal–Wallis *p* < 0.05) by a given diet. The remaining five types of diets not shown are: low‐carbohydrate diet, high‐fat and high sugar diet (HFHS diet), MRE, high‐gluten diet, and low‐calorie diet. (F) Diet reshapes the landscape of phage‐host interactions and lifestyles. From top to bottom, the panels show: (1) changes in virome α‐diversity (Shannon index); (2) the concordance of phage–bacterium abundance shifts, with red indicating concordant (same direction) and blue discordant changes; (3) the proportion of significantly altered phage–bacteria correlations (Spearman's *ρ*, *P*_adj_ < 0.05 with Benjamini–Hochberg correction); and (4) the proportion of phage lifestyle after dietary intake. Statistical significance was determined by the two‐sided Wilcoxon rank‐sum test: *p < 0.05, **p < 0.01, ***p < 0.001, ****p < 0.0001; comparisons without asterisks are not significant. MED, Mediterranean diet; EEN, Exclusive enteral nutrition; WTP, Whole‐grains, Traditional Chinese medicinal foods and Prebiotics diet; MRE, Ready‐to‐eat Meal; Urban, High urbanization diet.

To elucidate the response patterns of the gut virome to diet, we conducted a meta‐analysis of 6932 gut metagenomic datasets from 62 BioProjects with associated dietary records from 20 countries across Asia, the Americas, Europe, and Oceania. All datasets were categorized into two main groups based on intake characteristics, namely, “dietary types” and “dietary components.” This dataset encompasses 24 dietary types and 44 nutritional components (Figure 1A).

To dissect the factors shaping the human gut virome, we quantified the variance explained by host and environmental variables using permutational multivariate analysis of variance (PERMANOVA). Our analysis revealed that nutrition and diet were the primary drivers of variation in virome composition, explaining a substantially larger fraction of the variance (dietary types: *R* ^2^ = 0.14; nutritional components: *R* ^2^ = 0.20) than geography, health status, or medication use (Figure 1B and Figure S2A,B). Even after adjusting for age and disease status as covariates in multivariate models, diet remained the dominant driver of virome variation (*R* ^2^ = 0.078, *p* < 0.05), exceeding both age (*R* ^2^ = 0.005) and disease status (*R* ^2^ = 0.006) (Data S3). This dietary influence was mirrored in the bacteriome, suggesting widespread interactions between viruses and bacteria under dietary regulation. In further support of the pervasive regulatory role of diet, we observed that dietary intake promoted a convergence of the virome structure across individuals. Specifically, a comparison of inter‐individual Bray–Curtis distances showed a significant increase in community similarity following five of the seven dietary interventions (Wilcoxon test, *p* < 0.05; Figure S2C). Among the different dietary types, those that primarily modulated fat (*R* ^2^ = 0.74) and fiber (*R* ^2^ = 0.19) contents had a greater impact on the virome (Figure 1C), suggesting a strong shaping effect on the virome.

Given that the gut virome is primarily composed of phages and that phage–bacteria interactions are critical drivers of gut microbial homeostasis, we identified differentially abundant phage species in response to diet using linear discriminant analysis effect size (LEfSe) analysis applying a linear discriminant analysis (LDA) score threshold of >2. We found that diet‐responsive phages predominantly infected hosts belonging to the phyla Firmicutes and Bacteroidetes (Figure 1D and Data S3). Notably, 49.39% (41 out of 83) of the crAssphage members, which specifically infect Bacteroidetes, showed significant changes in abundance in response to dietary interventions (LEfSe analysis, LDA > 2, Kruskal–Wallis *p* < 0.05). Consistent with our PERMANOVA results, dietary types that modulated fat and fiber content were the most common drivers of these shifts in phage abundance (Figure S2D). Furthermore, the effect of diet on the phagenome showed specificity at the bacterial phylum level (Figure 1E). For example, a high‐fat diet induced changes in the abundance across a broad taxonomic range of phages, with hosts distributed across Firmicutes (36.36% of the phages affected), Bacteroidetes (50.00%), Actinobacteria (44.83%), and Proteobacteria (34.21%). In contrast, the shifts associated with Mediterranean, high‐fiber, and vegetarian diets primarily affected phages that infect Firmicutes and Bacteroidetes. Considering the similar impacts of vegetarian and high‐fiber diets on virome remodeling, we further compared their diet‐responsive phages. The observation that 23.9% of responsive phages were shared points to dietary fiber as a pivotal common driver, whereas the phage responses unique to the vegetarian diet likely stem from the distinct contributions of other plant‐derived constituents, such as polyphenols and plant proteins (Figure S2E). These findings suggest that different dietary types exert specific regulatory pressures on the composition and structure of the gut phagenome. This specificity was further corroborated by alterations in phage alpha‐diversity, with high‐fat, Mediterranean, and vegetarian diets leading to a significant increase in diversity (Wilcoxon test, *p* < 0.05; Figure 1F).

Further analysis revealed that dietary interventions remodeled the phage–bacteria interaction patterns (Figure 1F). Various diets, including high‐fat, Mediterranean, and Western‐type pattern (WTP) diets, induced co‐directional abundance changes in numerous phages and their respective bacterial hosts (Figure S3). Consequently, the correlations between phages and their specific hosts changed significantly. For instance, after a high‐fat diet, positive correlations were strengthened for phages and their corresponding hosts, such as *Lactococcus*, *Lactobacillus*, *Pseudomonas*, and *Klebsiella* (*P*_adj_ < 0.05 with Benjamini–Hochberg correction; Figure S4). Diet also appeared to influence phage lifestyles. A higher proportion of temperate phages (69.23%) was observed following a high‐fat diet, whereas the vegetarian diet favored virulent phages (75.00%). These findings suggest that diet regulates phage–bacteria interactions through multiple mechanisms, including modulating specific phage abundance and shifting phage lifestyles. This regulation has also been observed at the phylogenetic level. A phylogenetic tree constructed from 1,400 high‐quality phage genomes revealed non‐random clustering patterns associated with specific diets (Figure S5). For example, in samples from mice fed a high‐fat diet, phages belonging to the family *Myoviridae* and infecting Actinomycetota hosts clustered significantly within a specific clade, suggesting shared ecological adaptations or functional traits under the same diet. Additionally, phages exhibited phylogenetic clustering according to bacterial host categories and lifestyles, further supporting the notion that diet may regulate phage–bacteria interactions via selective enrichment of the phagenome.

### 2.2 Fucoidan Supplementation Counteracts High‐Fat Diet‐Induced Phage Proliferation

**FIGURE 2:**
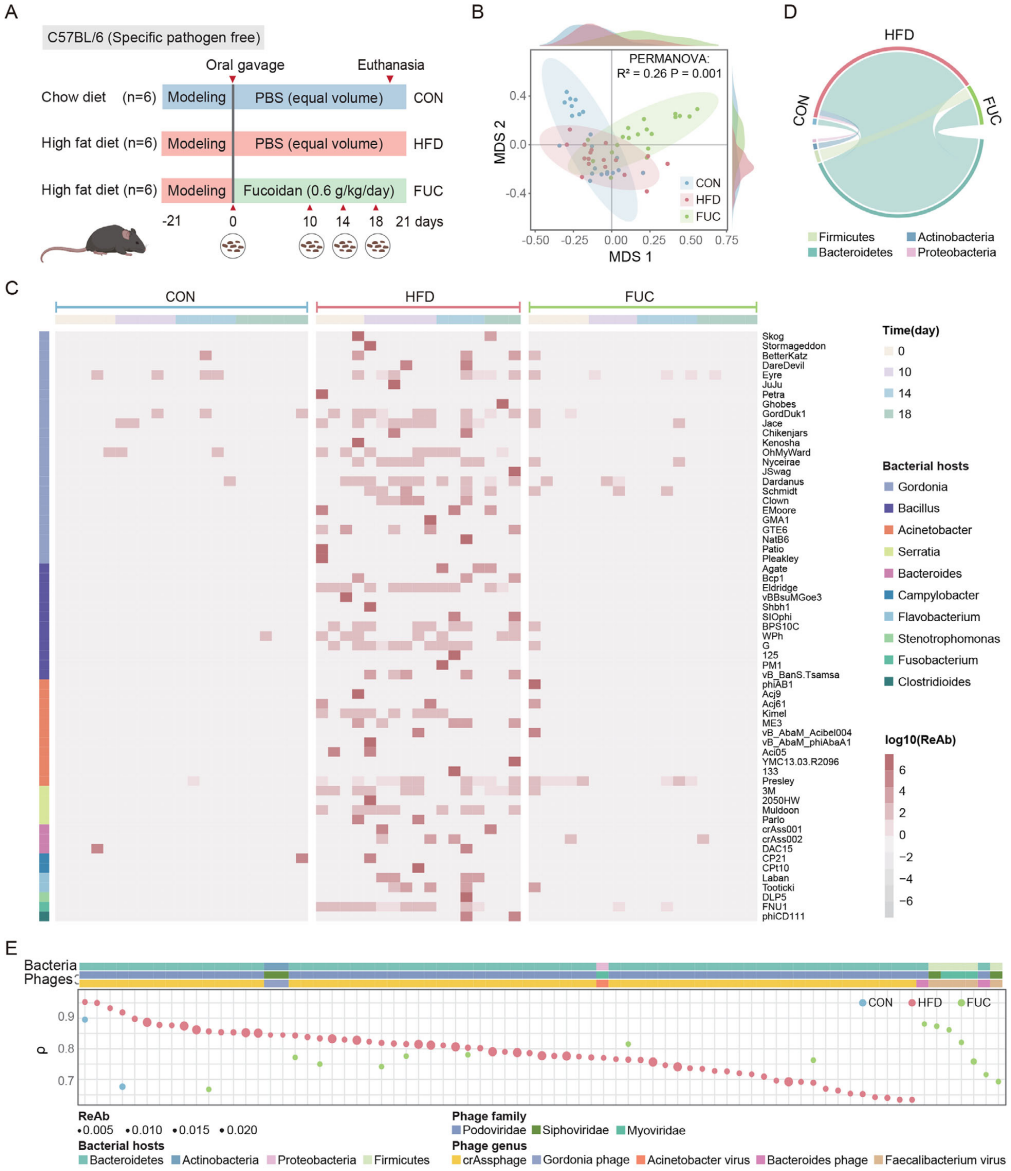
Fucoidan (FUC) supplementation counteracts high‐fat diet (HFD)‐induced phage proliferation. (A) Experimental design. Specific‐pathogen‐free (SPF) female C57BL/6J mice (*n* = 6 per group) were fed a control diet (CON), a high‐fat diet (HFD), or an HFD supplemented with fucoidan (FUC; 0.6 g/kg/day by oral gavage) during weeks 4–7. Fecal samples were collected on days 0, 10, 14, and 18 of the FUC intervention period. (B) Diet reshapes gut bacterial community structure. Non‐metric multidimensional scaling (NMDS) ordination (stress = 0.16, *n* = 57) of gut bacterial profiles based on Bray–Curtis dissimilarities. Points are colored by dietary group: CON (blue), HFD (red), and FUC (green). Ellipses represent 95% confidence intervals. Group separation was statistically significant (Adonis test, *p* < 0.001, 999 permutations). (C) Temporal dynamics of phages infecting diet‐associated bacteria (DABs). The heatmap illustrates the log‐transformed relative abundance (ReAb) of specific phage species over the intervention period. Phages (rows) are clustered by their predicted host genus, revealing diet‐driven shifts in abundance. (D) Taxonomic profile of phages associated with DABs. This chord diagram shows the taxonomic composition of phages (at the host‐phylum level) that were significantly correlated with DABs at day 10 of the intervention (Spearman's *ρ*, *P*_adj_ < 0.05 with Benjamini–Hochberg correction). (E) Phage‐DAB correlations are modulated by diet. The height of each point represents the Spearman correlation coefficient (*ρ*), its size scales with phage abundance, and its color indicates the dietary group. Only significant correlations (*p* < 0.05) are shown. Top color bars indicate the taxonomy of bacterial hosts (phylum), phage families, and phage species.

Although our meta‐analysis indicates that high‐fat diets and dietary fiber have a widespread and notable effect on shaping the gut virome, it is not possible to fully explain whether these diet‐induced changes are temporary fluctuations or stable shifts based solely on existing public data, as the available data primarily comprise cross‐sectional studies or longitudinal tracking of a single dietary component. Thus, we designed a longitudinal study using a mouse model to elucidate the patterns by which high‐fat diets and dietary fiber intake shape the gut virome. In addition to a high‐fat diet (HFD), we fed mice fucoidan (FUC), a dietary fiber that is not commonly studied in this context and was not included in our meta‐analysis (Figure 2A). During the 18‐day intervention period, we first observed that FUC supplementation significantly mitigated HFD‐induced obesity, as evidenced by a 22.01% reduction in mean body weight (Figure S6A; post‐hoc Tukey's HSD, *p* < 0.05). This physiological improvement was accompanied by significant remodeling of the gut microbiota composition (Figure 2B; Adonis test, *p* < 0.05). The changes in microbiota composition were time‐dependent, with intergroup separation progressively intensifying and FUC supplementation directing the microbiota along a trajectory distinct from that of HFD alone (Figure S7).

To determine whether the gut virome responded to these microbiota shifts, we first identified diet‐associated bacteria (DABs) using LEfSe analysis (LDA score > 2, Kruskal–Wallis *p* < 0.05). After 10 days of HFD feeding, we identified 21 bacterial genera with significantly altered abundances. In the FUC group, only six of these genera remained significantly different, indicating that FUC partially rescued the HFD‐induced dysbiosis (Figure S6B). Next, we tracked the abundance of the phages infecting these DABs. Across nearly all individuals and throughout the study period, the HFD group consistently exhibited higher phage abundances than those of the FUC‐supplemented and control groups. These results suggested that the HFD regimen triggered a proliferation of phages associated with DABs, leading to a stable high abundance that was reversed by FUC supplementation (Figure 2C).

To assess the impact of these dietary perturbations on phage–bacterial interactions, we calculated the correlations between the abundance of phages and their bacterial hosts. The HFD group displayed the highest number of significant phage–host correlations (Spearman's *ρ*, *P*_adj_ < 0.05, with Benjamini–Hochberg correction), which was predominantly linked to hosts from the Bacteroidetes phylum (1826 pairs). In contrast, FUC supplementation increased the number of correlations involving hosts from the Firmicutes phylum (Figure 2D and Figure S6C). These phylum‐level differential patterns were further validated through time‐series analysis (Figure S6D). The number of phage–bacterial correlations in the HFD group increased continuously throughout the intervention, particularly for Bacteroidetes phages, whereas the FUC group exhibited an initial decrease followed by recovery, with only Firmicutes phage–bacterial correlations showing sustained increases. Moreover, in both the HFD and FUC groups, the phages were strongly correlated with the DAB hosts. Phages infecting either Bacteroidetes (e.g., crAssphage) or Firmicutes hosts consistently showed high correlation coefficients (*ρ* ≥ 0.63) with their respective hosts (Figure 2E). Collectively, these findings demonstrate that both the HFD and FUC regimens intensify phage–bacteria interactions and confirm that the gut virome's response is highly diet‐specific.

### 2.3 High‐Fat Diet and Fucoidan Drive Reciprocal Transitions in Phage Lifestyles

**FIGURE 3:**
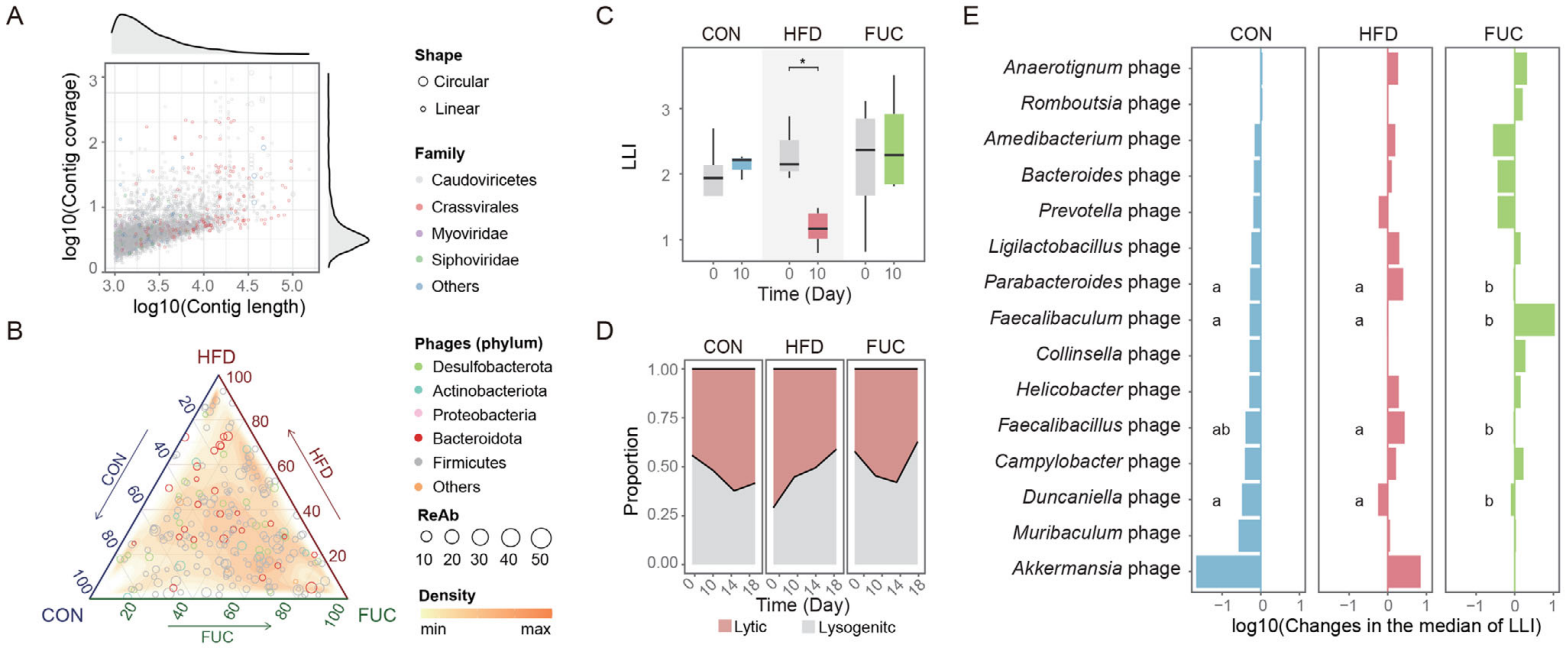
Lytic‐lysogenic switch of phages induced by HFD and FUC dietary intake. (A) Characterization of viral contigs. This plot displays the length and sequencing coverage for each assembled viral contig (*n* = 53 584), providing an overview of the sequencing data quality. Colors denote the assigned viral family, and larger circles specifically mark contigs identified as circular. (B) The diet‐driven shift in phage lifestyles after 10 days of intervention. The position of each vOTU reflects its relative change in the lytic‐lysogenic index (LLI) across the three dietary groups (vertices: CON, HFD, FUC), calculated as log_10_(LLI_day10/LLI_day0 + 1), where LLI = | log_2_(virulent vOTU abundance+0.1 / temperate vOTU abundance+0.1) |. Point size is proportional to the vOTU's median relative abundance, and color indicates the phylum of its bacterial host. Density shading reflects the distribution of vOTUs (*n* = 21 029). (C) Diet alters the overall balance of phage lifestyles. These box plots display the LLI at baseline (Day 0) and after 10 days of intervention, which elements are defined as: center line, median; box limits, upper and lower quartiles; whiskers, 1.5× interquartile range. Statistical significance was determined by the two‐sided Wilcoxon rank‐sum test (*n* = 6 per group): *p < 0.05; comparisons without asterisks are not significant. (D) Temporal dynamics of phage lifestyles during dietary intake. (E) Magnitude and direction of lifestyle shift in the phage of DABs. Each bar represents the log10‐transformed fold change (Day 10 vs. Day 0) in the median of LLI for all phages associated with a specific host genus. LLI was calculated as the median of (virulent phage abundance + 1) / (temperate phage abundance + 1) across all phages infecting that host. Positive values indicate a shift toward lytic dominance, while negative values indicate a shift toward lysogenic dominance. Significant differences among diet groups for each phage (*n* ≥ 5) were identified using a Kruskal–Wallis test, followed by Dunn's post‐hoc test for pairwise comparisons with *p*‐values adjusted using the Benjamini–Hochberg method. For each phage, bars labeled with different letters indicate a statistically significant difference between groups (*p* < 0.05); bars without letters are not significantly different. LLI is defined as the abundance ratio of virulent to temperate phages. CON, control diet; HFD, high‐fat diet; FUC, HFD with fucoidan supplementation.

Since shifts between lysogenic and lytic life cycles may be a key driver of phage–bacteria interactions, we next investigated how HFD and FUC supplementation modulate phage lifestyle transitions. From our metagenomic assemblies, 53 584 viral contigs were identified. Taxonomic and lifestyle analyses indicated that the majority of these contigs belonged to the class *Caudoviricetes*, with the families *Siphoviridae* and *Myoviridae*, and the order *Crassvirales* (Figure 3A). These contigs were clustered into 21 029 viral operational taxonomic units (vOTUs) with 85% sequence identity. To assess phage lifestyle dynamics, we established a lytic–lysogenic index (LLI), which is defined as the abundance ratio of virulent to temperate phages. By comparing the changes in LLI from day 0 to 10 after 10 days of dietary intervention, we found that both HFD and FUC supplementation significantly drove shifts in phage lifestyles (Figure 3B and Figure S8A; Wilcoxon test, *p* < 0.05). Notably, this transition was more pronounced in phages infecting Bacteroidetes hosts under the HFD regimen (Figure S8B; Wilcoxon test, *p* < 0.05).

At the baseline, the phage community was predominantly in a lytic state. However, HFD intake induced a community‐wide shift toward lysogeny, as reflected by a significant 24.53% decrease in the average LLI from day 0 to day 10 (Wilcoxon test, *p* < 0.05). This shift was further supported at the genomic level by the reduced abundance of lysis‐associated genes (holins and lysins) in HFD‐treated phages compared to that in FUC‐supplemented phages on day 10 (Figure S8C), indicating that HFD attenuates the capacity of phages to regulate the gut microbiota through lysis of their bacterial hosts. The trend in the FUC‐supplemented group was similar to that in the control group, indicating that FUC partially counteracted the HFD‐induced lifestyle transition (Figure 3C,D). In contrast, when we focused specifically on phages infecting DABs, the opposite trend was observed. While these phages tended toward lysogeny in the control group, HFD intervention prompted a significant shift toward a lytic state, with the average LLI increasing by 8.87 times (Wilcoxon test, *p* < 0.05). This suggests that diet exerts a distinct regulatory pressure on phages infecting DABs, independent of the overall phage community (Figure S8D,E). For instance, *Parabacteroides* phages shifted toward a lytic lifestyle under the HFD, a change that was sustained over time, whereas FUC supplementation significantly attenuated this HFD‐driven transition (Figure 3E and Figure S8F,G; Dunn's post‐hoc test, *p* < 0.05). Furthermore, FUC supplementation induced a transient lytic shift in *Faecalibaculum* and *Anaerotignum* phages, which primarily infect Firmicutes. This likely contributed to the observed reduction in the abundance of genera of the Firmicutes in the FUC group, providing further evidence that the alternation of high‐fat and polysaccharide diets can drive lifestyle transitions in specific phages.

### 2.4 Fucoidan Restores Phage‐Mediated Horizontal Gene Transfer Suppressed by High‐Fat Diet Intake

As another critical form of phage–bacterial interaction, phage‐mediated HGT is likely to be altered by diet‐induced shifts in phage lifestyles. Therefore, we sought to determine the effects of HFD and FUC interventions on HGT. By identifying potential HGT fragments in bacterial genomes and aligning them against phage genomes, we detected 7115 HGT events. Of these, 476 were potentially phage‐mediated, with 46 involving phages that infect DABs.

**FIGURE 4:**
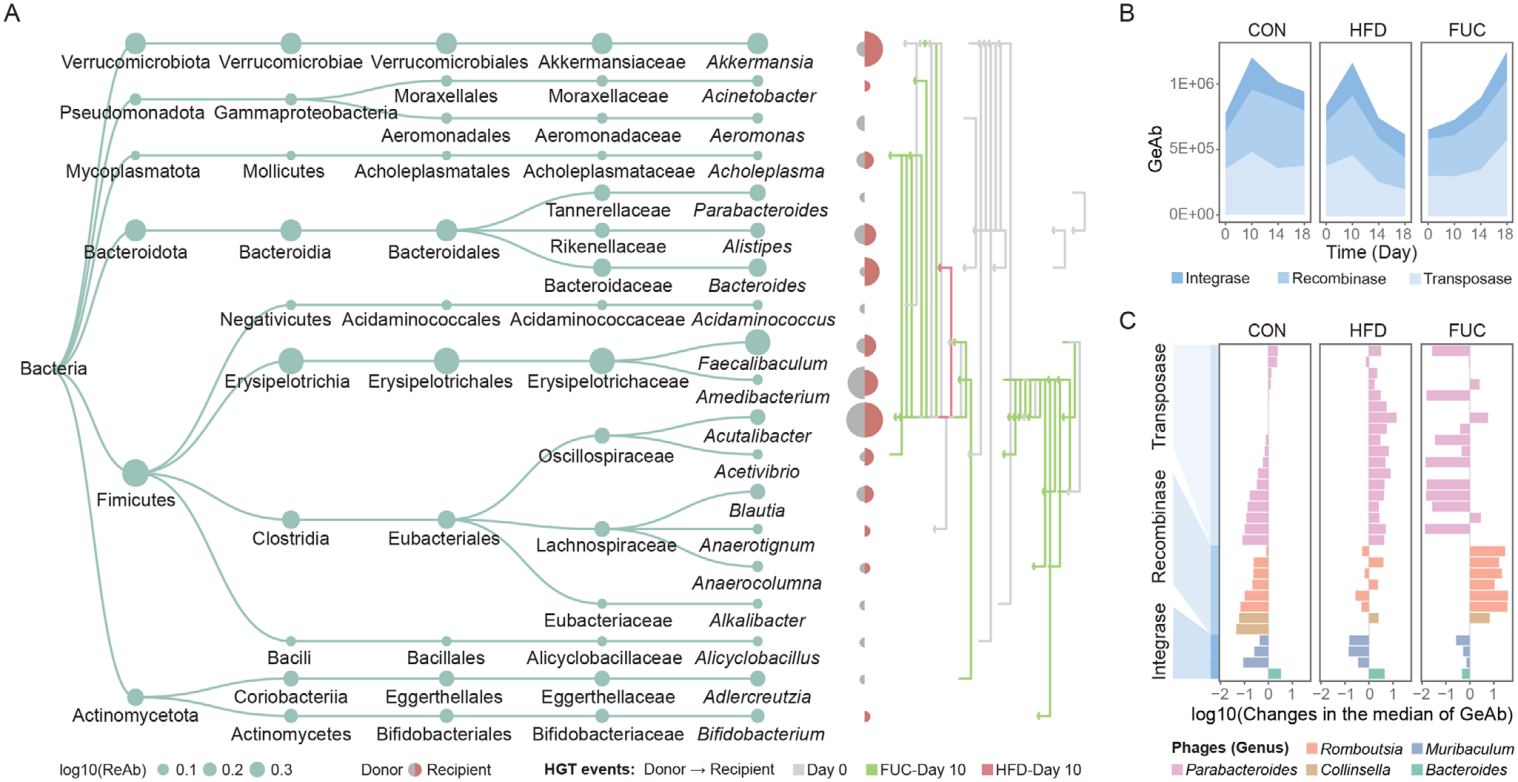
FUC restores phage‐mediated HGT reduced by HFD intake. (A) Distribution of phage‐mediated HGT events under different diets. This panel includes a phylogenetic map of bacterial hosts (left; node size proportional to host abundance), a summary of HGT donor and recipient proportions (middle), and a network diagram illustrating directional HGT events (right; arrows indicate direction, lines colored by group). (B) Temporal profiling of HGT‐related gene abundance in phages during dietary intake. (C) Diet‐driven changes in HGT‐related gene abundance in phages of DABs. The bar chart shows the log10‐transformed fold change (Day 10 vs. Day 0) in the median normalized abundance (GeAb) of HGT‐related genes for individual phage contigs. Each bar represents a phage contig, colored by its bacterial host. Positive values (log_10_[Day10‐Day0], Day10>Day0) indicate an increase in abundance; negative values (‐log_10_[|Day10‐Day0|], Day10<Day0) indicate a decrease. Sample size *n* = 6 mice per group. CON, control diet; HFD, high‐fat diet; FUC, HFD with fucoidan supplementation.

Relative to their respective baselines, HFD intake led to an 80% reduction, and FUC supplementation resulted in an 8.5‐fold increase in phage‐mediated HGT frequency. However, there was minimal overlap in donor–recipient pairs between FUC‐associated HGT events and baseline HGT events. This suggests that while FUC restored the HGT levels suppressed by HFD, it did so by inducing a novel set of HGT events rather than reverting to the original state (Figure 4A). Functional annotation of these novel HGT genes revealed diverse categories, including nucleotide metabolism, ribosome function, and stress defense, suggesting that FUC‐enhanced HGT promotes broader community adaptation (Data S6). We found that phage‐mediated HGT events occurred most frequently within the Firmicutes and Bacteroidetes phyla, indicating that phages promote frequent genetic exchange within these bacterial lineages under dietary perturbation. During these events, different bacteria played distinct roles: *Akkermansia* and *Bacteroides* were more likely to act as recipients, *Amedibacterium* was more inclined to be a donor, and *Acutalibacter* could serve as both.

To explore the underlying mechanisms, we searched for HGT‐related enzymes in the phage sequences, including integrases, recombinases, and transposases. We found that the abundance of these three enzyme types increased continuously over the intervention period only in the FUC‐supplemented group, partially supporting the potential of FUC to facilitate phage‐mediated HGT (Figure 4B,C). Among the phages infecting DABs, *Parabacteroides* phages possessed the most diverse transposase genes, with their relative abundances showing opposite trends under HFD and FUC supplementation (Figure 4C and Figure S9A). Therefore, we hypothesized that *Parabacteroides* phages are major contributors to the FUC‐driven restoration of HGT levels.

### 2.5 Diets With High Fat and Fucoidan Enhance Phage Auxiliary Metabolic Functions

**FIGURE 5:**
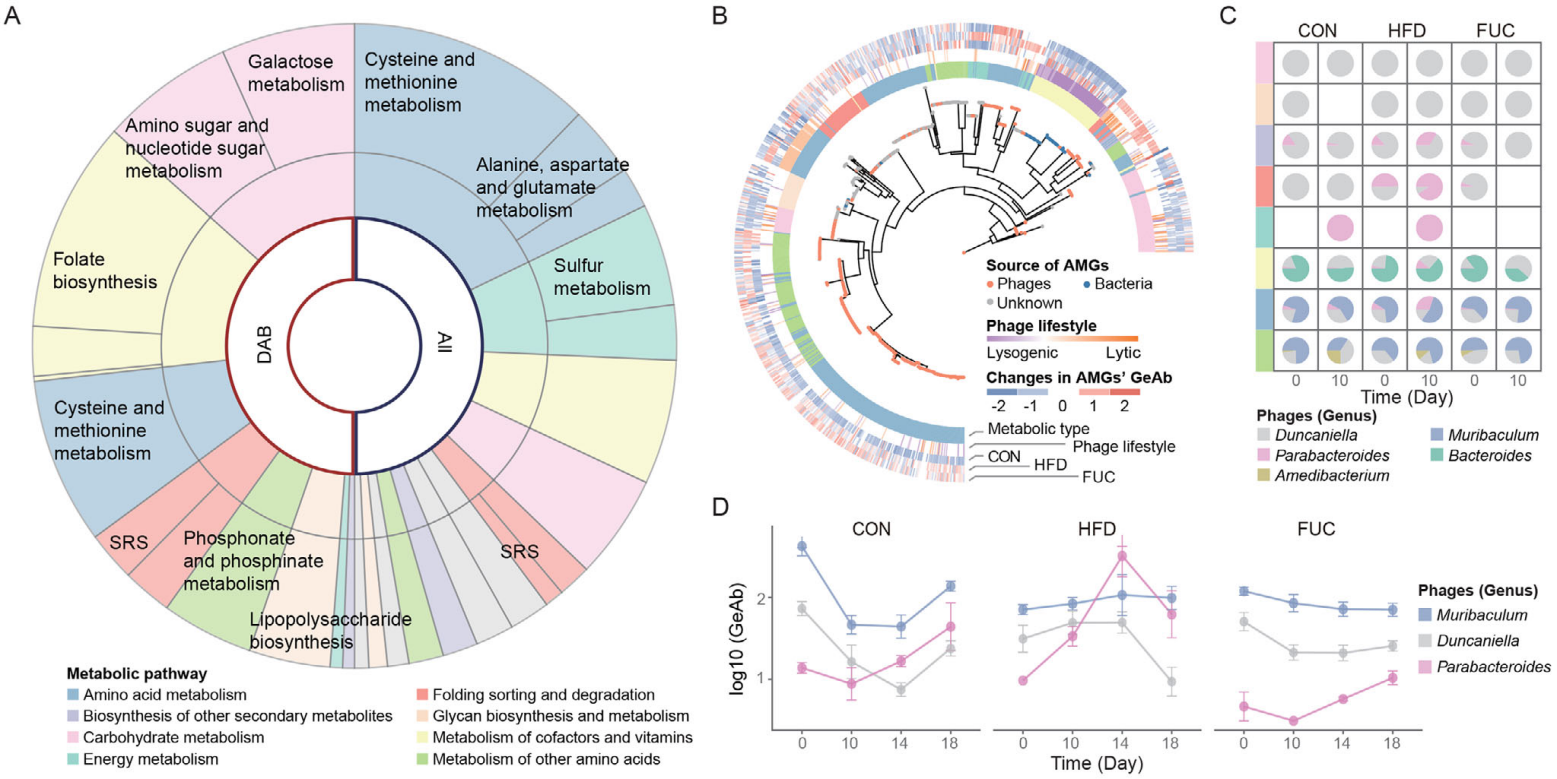
Enhanced auxiliary metabolic functions of phages under HFD and FUC dietary intake. (A) Diet‐driven changes in the AMG functional profile. The comparison shows the abundance of AMGs in phages infecting DABs (left) versus all bacterial hosts (right), grouped by metabolic pathway. (B) Integrated view of AMG evolution, function, and response to diet. The central tree shows the phylogeny of cross‐kingdom AMGs (see Methods). Node colors indicate the source of AMGs and homologs. The concentric rings, from inner to outer, connect each AMG to: (1) its assigned metabolic categories (KEGG Level 2); (2) the lifestyle of its carrier phage; and (3) its abundance change (log10‐fold change, Day 10 vs. Day 0) under each diet. Positive values (log_10_[Day10‐Day0], Day10>Day0) indicate an increase in abundance; negative values (‐log_10_[|Day10‐Day0|], Day10<Day0) indicate a decrease. (C) The contribution of AMGs abundance from phages of DABs (Day 10 vs. Day 0). Rows represent metabolic categories of AMGs. (D) Temporal dynamics in the normalized abundance (GeAb) of amino acid metabolism‐related AMGs from phages of DABs. Data are presented as mean ± s.e.m. Sample size *n* = 6 mice per group. CON, control diet; HFD, high‐fat diet; FUC, HFD with fucoidan supplementation; SRS, sulfur relay system.

HFD and FUC interventions alter the frequency of phage‐mediated HGT, which provides an opportunity for phages to acquire exogenous AMGs via genetic exchange. To explore the acquisition of phage AMGs following dietary intake, we identified 3174 AMGs in our phage genomes. In contrast to the overall phage community, where AMGs for amino acid metabolism were dominant, phages infecting DABs showed increased proportions of AMGs related to carbohydrate metabolism and the metabolism of cofactors and vitamins (Figure 5A). This indicates that HFD and FUC caused phages to carry more AMGs with specific functions, which may potentially enhance the corresponding metabolic capacities of the gut microbiota.

We further examined the similarities between phage AMGs and bacterial genomes and found that their homologous proteins were more frequently of viral rather than bacterial origin (Figure 5B). We also observed that phage AMGs exhibited distinct preferences driven by both dietary interventions and phage lifestyle. For phages infecting DABs, AMGs related to cofactor and vitamin metabolism were more prevalent in the lysogenic state (17.20%), whereas AMGs involved in amino acid metabolism were predominant in the lytic state (54.91%). This trend was further validated in the phylum Bacteroidota across all phages, where 38.78% of the AMGs carried by lytic phages were associated with amino acid metabolism, and lytic phages contributed 52.83% of the total amino acid metabolism AMGs within this phylum. The abundance of these amino acid metabolism AMGs increased following HFD (142.00% increase on day 10, 584.20% on day 14, and 156.67% on day 18, relative to day 0). Pathway‐resolved analysis further identified specific AMGs driving this pattern, including α‐aminoadipate semialdehyde synthase (K14157) and lysine decarboxylase (K01582) in lysine degradation, asparagine synthetase (K01953), MTA/SAH deaminase (K12960), and CDP‐diacylglycerol‐serine O‐phosphatidyltransferase (K17103), all of which exhibited marked HFD‐induced upregulation, which was reversed by FUC (Figure S9B). FUC supplementation attenuated the HFD‐induced enrichment of AMGs for amino acid metabolism (7.18% decrease on day 10, 30.91% on day 14, and 32.40% on day 18, relative to day 0). A similar trend was observed for AMGs associated with energy metabolism (Figure S9C). Notably, in both high‐fat‐containing diet groups, the abundance of AMGs related to lipid metabolism significantly increased in the overall phage community, irrespective of FUC supplementation. This suggests that phages may augment the corresponding host metabolic pathways to enhance dietary adaptation.

Across these metabolic pathways, we identified *Parabacteroides* phages as the primary contributors of AMGs for energy, amino acid, cofactor, and vitamin metabolism (Figure 5C). Furthermore, in *Parabacteroides* phages, the abundance of AMGs related to amino acid and energy metabolism increased following the HFD intervention. In contrast, FUC supplementation reversed this effect, causing their abundance to align with that of the control group in a sustained manner (Figure 5D), demonstrating that FUC counteracted HFD‐induced alterations in these phage AMGs. These findings establish that high‐fat and polysaccharide‐rich diets modulate specific phage–bacterial metabolic processes via AMGs, thereby strengthening their interplay. *Parabacteroides* phages, in particular, appear to play a pivotal role in this dynamic.

### 2.6 Bacteroidetes Phages Respond to Diverse Polysaccharides and Maintain Stable Interactions With Bacterial Hosts

**FIGURE 6:**
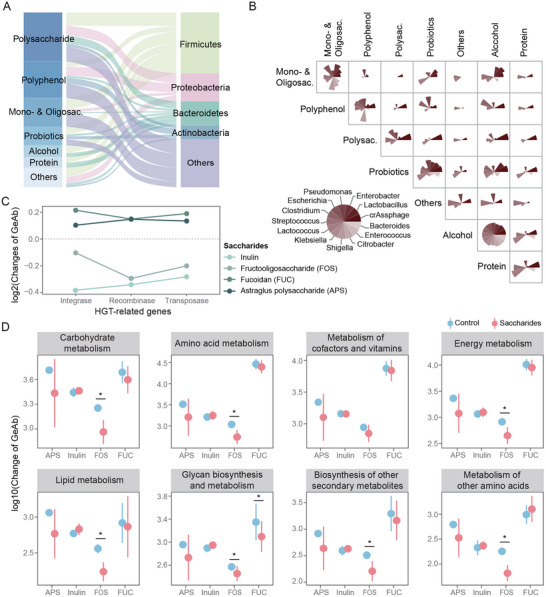
Broad responsiveness and interactions of phages to dietary saccharides. (A) Responses of phages across diverse dietary components. This alluvial plot illustrates the proportion of phage taxa (grouped by host phylum) that significantly change in abundance in response to different dietary components (LEfSe, LDA score > 2, Kruskal–Wallis *p* < 0.05). The width of each flow is proportional to the percentage of responding phages within a phylum. (B) Shared phage–bacteria interactions across different dietary components. Each sector represents a bacterial genus and its associated phages. The area of each sector is proportional to the number of shared significant correlations (Spearman's *ρ*, P_adj_ < 0.05 with Benjamini–Hochberg correction) observed for the same phage‐bacterium pair across at least two distinct dietary conditions. Shared associations are defined as phage‐bacteria pairs that are significantly correlated in more than one dietary group. (C) Saccharide‐induced changes in phage HGT‐related genes. It plots the log2‐fold change in the mean normalized abundance (GeAb) of HGT‐related genes (integrases, recombinases, and transposases) in dietary intervention groups relative to their respective controls (HFD). (D) Saccharide‐induced changes in phage AMGs abundance. It plots the log10‐fold change in the GeAb of AMGs. Data are presented as mean ± s.d. Statistical significance was determined by the t‐test: *p < 0.05; comparisons without asterisks are not significant. Mono‐ & Oligosac., Mono‐ & Oligosaccharides; Polysac., Polysaccharides.

Building on the observation that FUC can partially counteract the dysbiosis of the gut virome and phage–bacteria interactions induced by high‐fat intake, we explored the specific patterns of virome modulation by other dietary polysaccharides and their heterogeneity. By analyzing the collected metagenomic dataset of 6932 samples, we confirmed that polysaccharides are a primary nutritional driver of phage abundance changes (Figure S9D), consistent with our FUC supplementation results. These changes preferentially involved phages infecting Firmicutes (Figures 2D and 6A).

crAssphages, which infect Bacteroides bacteria, dominated the network of phage–bacteria interactions driven by six distinct polysaccharides, including inulin, astragalus polysaccharides (APS), and resistant starch. These phages accounted for the vast majority of the significant correlations with host bacteria (14 012 pairs, 98.67%), far surpassing all other phages, and most of these associations remained significant across multiple dietary component intakes. This indicated that crAssphages exhibited a broad response to various polysaccharides and maintained relatively stable interactions with their bacterial hosts (Figure 6B). Dietary interventions with polysaccharides led to a decrease in the proportion of lytic phages, favoring a general shift toward lysogeny (66.67%, Figure S9E). In contrast, oligosaccharides produced the opposite effect, highlighting the functional heterogeneity of the dietary components.

The contrasting effects of HFD and FUC on phage–bacterial interactions prompted us to investigate whether different saccharides could affect HFD‐induced alterations. Extended analysis revealed that APS, similar to FUC, attenuated the increase in the abundance of HFD‐enriched phages and increased the number of phage–bacteria correlations. Furthermore, both APS and inulin showed significant differences in LLI compared to the HFD group, indicating that different polysaccharides possess the capacity to modulate phage lifestyle transitions (Figure S10). Indeed, the profiles of the HGT‐related enzymes varied markedly among the different saccharides. Fructooligosaccharides (FOS) and inulin downregulated genes encoding integrases, recombinases, and transposases. In contrast, only APS mirrored the effect of FUC, concurrently upregulating the abundance of genes encoding all three enzymes (Figure 6C). This mechanistic divergence extended to AMGs. Inulin upregulated the abundance of AMGs in eight metabolic pathways, whereas FOS and APS downregulated them (Figure 6D). Despite these differences, the metabolic pathways of carbohydrates, amino acids, cofactors, and vitamins were consistently altered across all the saccharide interventions. Collectively, these distinct responses in HGT‐related enzymes and AMGs demonstrate that while different polysaccharides may modulate the gut virome in similar ways, they employ distinct mechanisms to reverse the functional and relational shifts in phage–host interactions caused by HFD.

## 3 Discussion

This study presents a large‐scale meta‐analysis of diet‐associated metagenomes, providing a systematic characterization of the gut virome's response to dietary intake. We conducted a broad assessment of how dietary types and nutritional components shape the gut virome. Using an alternating high‐fat and polysaccharide (FUC) diet design, we dissected the dynamic mechanisms of phage lifestyle switching, the functional enrichment of AMGs, and the resulting phage–host interactions. Our findings revealed that phages exhibit both relative independence and mediating roles in the diet‐driven modulation of the gut microbiota, particularly in high‐fat and polysaccharide interventions. These results provide a high‐resolution landscape of the interplay between diet, the virome, and bacterial hosts, establishing a new framework for understanding how diet regulates gut homeostasis via the virome and laying the groundwork for virome‐targeted precision nutritional strategies.

While previous studies have established a correlation between diet and virome variation, with similar diets fostering convergent viromes [23, 24, 25, 26], these investigations were often confined to short‐term interventions with single dietary patterns in small cohorts [24, 25] or correlational analyses with individual food items [26]. They lacked a systematic comparison of the perturbation intensity of diverse dietary types or nutritional components on the virome. Even in studies involving multiple dietary types [23], the explanatory power of diet versus other host factors has not been quantified, leaving it unclear whether different interventions have a selective preference for specific viral taxa. Our research addresses these gaps, demonstrating that the influence of dietary types and nutritional components on the virome is stronger than that of geography or health status. At least half of the dietary interventions that we examined significantly altered the viral community structure. Furthermore, individuals fed similar diets exhibited convergent viromes, with distinct diets selectively enriched for specific viral taxa. This study provides, from the largest diet‐associated virome analysis to date, evidence that diet exerts a powerful, widespread, and directional shaping force on the human gut virome.

Because the gut virome is predominantly composed of phages [14, 27] and has an obligate dependence on bacterial hosts, the prevailing view is that the virome's response to external perturbations largely follows or even mirrors that of the bacteriome [28, 29, 30, 31]. However, our findings challenge this view by revealing a more complex and partially independent dynamic of the diet‐shaped virome. Our multivariate analysis revealed that diet was the strongest shaping factor for the virome but not for the bacteriome. In our experimental validation, regardless of fluctuations in the abundance of DABs, the abundance of their phages remained consistently higher in the HFD group than in the FUC‐supplemented and control groups, which was sustained throughout the 3‐week intervention. This indicates that the virome's response to diet is not merely a passive reflection of the bacteriome's response; rather, it is more sensitive and direct. This is also consistent with observations in inflammatory bowel disease (IBD), where the gut virome (particularly phage communities) shows disease‐specific remodeling that may not be fully explained by concurrent shifts in the bacterial community [32]. Heightened sensitivity was also observed in a diet with high refined sugar and milk fat, where the virome showed impaired resilience compared to the bacterial community [33], suggesting that the virome is a highly tractable target for dietary modulation. Even if some phage members change secondary to their bacterial hosts’ dietary responses [34, 35], the cascading effects of ecological regulation cannot be ignored [36], as the primary role of phages is to drive bacterial succession as bacterial predators [37]. This hypothesis has been mechanistically elucidated through experimentation [38]: dietary whey protein can induce specific phages (e.g., AkkZT003P) to lyse their host bacteria (*Akkermansia muciniphila*), thereby altering the downstream microbial interaction network and alleviating intestinal inflammation, providing the direct evidence that a single dietary component can reshape the gut microbial community through phage‐mediated mechanisms. From this perspective, our discovery of the widespread, potent, and convergent shaping effect of diet on the virome highlights the importance of the gut virome in dietary interventions for gut microbiota and health.

The HFD has been proposed to exacerbate obesity by acting on the virome, either indirectly by affecting bacterial hosts susceptible to phage predation or directly by influencing phage induction and activity [39]. While natural polysaccharides can mitigate HFD‐induced obesity by modulating the microbiota and its metabolites [40] and low‐fat, plant polysaccharide‐rich diets can promote shifts in the lifestyle of phages [22], the role of the virome in the reversal of HFD‐induced dysbiosis by polysaccharides remains ambiguous. Despite abundant research data on HFD or polysaccharide supplementation alone, the absence of time‐series data in most studies has obscured the highly dynamic nature of the phage–bacteria interplay [41], leaving the functional role of phages in this process unresolved. To address this gap, we conducted a time‐series animal experiment using fucoidan, a polysaccharide not included in previous meta‐analyses, as the intervention component. Unlike well‐studied polysaccharides, such as inulin, the effect of fucoidan on the gut virome remains largely unexplored, which allowed us to generate novel data and test the generalizability of the observed response patterns. Moreover, fucoidan is distinguished from terrestrial plant polysaccharides by its unique structural features, including sulfate groups and a fucose‐rich backbone. Differences in structural properties and degradation pathways may selectively enrich distinct bacterial populations, thereby shaping different phage–host dynamics. Our experiments also demonstrated its ability to alleviate HFD‐induced obesity in mice (Figure S6A), making it an ideal candidate to validate whether polysaccharides can reverse HFD‐induced dysbiosis. By collecting time‐series metagenomic data during HFD/FUC alternation, we elucidated the dynamic patterns of phage responses to dietary perturbations and their interactions with bacteria, revealing the pivotal role of *Bacteroides* phages in this process and deepening our understanding of how polysaccharides influence the phage–host interplay and counteract HFD‐induced microbial dysbiosis.

When interpreting these interactions, the potential direct effects of fucoidan on phages should also be considered. Sulfated polysaccharides can bind to viral particle surfaces through electrostatic interactions owing to their negatively charged sulfate groups, thereby limiting viral transmission [42]. As a typical sulfated polysaccharide, fucoidan has demonstrated antiviral activity against various enveloped viruses (such as HIV [43], HSV [44], and RSV [45]), primarily by inhibiting viral adsorption and blocking viral entry into host cells [43], with activity positively correlated with sulfate group content [46]. Although most gut bacteriophages are non‐enveloped viruses and their abundance fluctuations are generally regarded as indirect reflections of host bacterial changes, biophysical evidence indicates that the capsid surface charges of non‐enveloped phages (such as PP7 [47] and MS2 [48]) exhibit high pH dependence and complex heterogeneous distribution patterns, providing a theoretical basis for direct interactions between fucoidan and phages under specific microenvironmental conditions. Furthermore, fucoidan undergoes partial degradation in the upper gastrointestinal tract, resulting in reduced molecular weight, while its primary structure remains stable in the intestine [49, 50]; lower‐molecular‐weight sulfated polysaccharides typically exhibit stronger antiviral activity, possibly attributable to their higher bioavailability and more effective binding to viral targets [51, 52], suggesting that the likelihood of direct interactions between digested fucoidan and phages may be further increased. Therefore, dietary modulation of the gut virome may not merely be a secondary consequence of bacteriome changes, with direct physicochemical interactions between polysaccharides and phages potentially representing an independent regulatory pathway.

Under the inflammatory conditions induced by HFD intake [53], phages have been shown to favor a lysogenic state to coexist with their bacterial hosts, leading to an increase in lysogenized bacteria [22], with over 55% of the strains undergoing lysogenic conversion within 3 days [54]. This community‐wide shift toward lysogeny echoes the “piggyback‐the‐winner” (PtW) dynamics described in environmental virology [55]. The PtW model suggests that under conditions of high host density and energy surplus, similar to the nutrient‐dense gut environment created by an HFD, temperate phages prefer to coexist with their hosts via lysogeny rather than killing them [56]. These lysogenic phages can encode AMGs to modulate host physiology, improving survival and maintaining overall community stability over broader host ranges [57]. However, our study found that while HFD induced a general shift toward lysogeny for most phages, those infecting DABs exhibited an enhanced lytic trend (Figure 3C,D and Figure S5C,D), suggesting that the dietary regulation of phage lifestyles is bacterial host‐specific. DABs such as *Parabacteroides* might be more sensitive to HFD‐induced environmental stress (e.g., reactive oxygen species), activating an SOS response that induces integrated prophages to enter the lytic cycle [58, 59]. This stress‐induced lytic switch is also evident in patients with IBD, where oxidative stress in the inflamed gut triggers widespread prophage induction [60]. The resulting lytic phages, which often carry AMGs that promote replication, can hijack the core metabolism of bacterial hosts for rapid self‐propagation [61], thereby evading coextinction with a stressed host. A similar phenomenon was observed in fructose‐rich diets, which can activate the SOS response and the lytic release of prophages [62, 63]. Upon FUC supplementation of the HFD, we observed not only a specific attenuation of the lytic shift in *Parabacteroides* phages but also a transient activation of a lytic shift in Firmicutes phages (Figure 3E). This differential modulation confirms that phages dynamically adopt distinct survival strategies in response to diet‐induced environmental pressures and host characteristics [64]. Compared to traditional induction methods, such as UV radiation or mitomycin C treatment [65], dietary components (e.g., FUC, whey protein [38]) offer a safe and tractable means to modulate phage life cycles with high specificity. This approach allows for the precise regulation of pathogenic bacteria without disrupting overall microbial homeostasis. Coupled with the fact that dietary viruses can colonize the human gut and maintain healthy gut viral ecology [57], dietary intervention has emerged as a safe and highly controllable strategy for phage‐targeted therapies of metabolic disorders.

In this study, phages of DABs showed an enrichment in AMGs for the metabolism of cofactors and vitamins in the lysogenic state. This likely reflects the adaptive need for the bacterial host to enhance energy metabolism and defend against oxidative stress in an HFD‐induced gut environment. Cofactor metabolism supports key enzymatic activities [66], whereas the synthesis of vitamins (e.g., B‐group vitamins) can enhance the metabolic stability and colonization capacity of the bacterial host, thereby improving glucose and lipid metabolism [67]. Concurrently, we found that the HFD enriched AMGs related to amino acid metabolism, especially in *Parabacteroides*, a trend that could be reversed by FUC supplementation. This may reflect metabolic pathway remodeling following the reduction of dietary fiber and other carbon sources caused by HFD. *Parabacteroides* can degrade complex polysaccharides [68]; however, its conventional energy acquisition pathways may be restricted by an HFD, prompting the enrichment of phage‐carried AMGs associated with amino acid metabolism as a means to adapt to nutritional limitations. Specifically, the upregulation of α‐aminoadipate‐semialdehyde synthase (K14157) suggests that phages may assist bacterial hosts in funneling lysine into the TCA cycle to maintain ATP generation [69], potentially compensating for energy deficits under carbon scarcity. Meanwhile, HFD‐induced bacterial membrane lipid stress [70] and oxidative damage [71] may be mitigated by phage‐mediated upregulation of CDP‐diacylglycerol‐serine O‐phosphatidyl transferase (K17103) and MTA/SAH deaminase (K12960), which provide membrane phospholipid biosynthesis [72, 73] and oxidative defense functions [74], respectively. Conversely, FUC supplementation created conditions for restoring carbohydrate‐based metabolic patterns by providing utilizable polysaccharides and alleviating HFD‐induced inflammation [75] and oxidative stress [76], thereby reducing the expression demand for these phage‐encoded AMGs. Notably, while the enriched AMG functions under HFD conditions enhance bacterial adaptation to lipid overload and favor phage reproduction, they may also drive host liver utilization of amino acids for fatty acid synthesis, potentially exacerbating conditions such as steatohepatitis [77]. Although potentially bacterial host‐specific, these observations offer critical insights into the patterns by which phages assist their bacterial hosts in response to dietary regulation.

Although bacterial phylum‐level changes [78] and metabolic responses at the functional level of specific phages show similarities across mammalian hosts, the response patterns of the gut virome to diet still depend on the host and its physiological state. HFD intervention in mice can stimulate the proliferation and stable maintenance of phages infecting DABs, transiently increasing virome α‐diversity [35] and significantly altering community structure [79]. Similarly, human viromes converge following the same dietary intervention [23], and specific phages are enriched in patients with T2D [80]. However, these patterns are not universal; for instance, HFD directly suppresses specific *Streptococcus* phages in a porcine model [81]. Regarding phage lifestyles, our study and other studies in mice showed that an HFD drives the enrichment of temperate phages in healthy animals [20, 22, 35]. In healthy humans, lytic phages are predominant [2], whereas a shift to the lysogenic state is observed in diseases such as IBD [60] and type 1 diabetes mellitus [82]. However, the response patterns of human gut phages after dietary interventions remain poorly understood, with observations limited to reduced food‐borne virus abundance in individuals with metabolic and inflammatory diseases [57]. Therefore, although mouse models reveal the critical mechanisms of diet–phage–bacteria interactions [38], their universality across different hosts, especially in human disease states, requires further validation. As a hypothesis‐generating foundational study, we elucidated core ecological principles using animal models, providing a testable framework for potential causal relationships among specific dietary components, phage lifestyle transitions, and host health outcomes. Establishing these causal links requires future targeted experiments using germ‐free animal models and controlled human trials, which would allow for a more accurate assessment of the clinical transformation potential of dietary interventions for the virome.

## 4 Conclusion

This study systematically mapped the regulatory patterns of dietary intake on the gut virome across a wide array of dietary types and nutritional components, elucidating the pivotal role of phages in mediating diet–microbiota interactions. We found that the high‐fat and polysaccharide diets exerted the strongest influence, intensifying the interplay between phages and their bacterial hosts. Specifically, HFD enhanced phage–bacteria interactions, drove the phages of DABs toward a lytic cycle, and enriched AMGs related to amino acid metabolism, which could exacerbate metabolic dysbiosis in the gut microbiota. Conversely, polysaccharide supplementation reversed HFD‐induced dysbiosis and promoted phage‐mediated HGT. Throughout the dynamic re‐equilibration of the microbiota during the HFD–polysaccharide transition, *Bacteroides* phages likely played a key role in responding broadly to various polysaccharides while maintaining stable interactions with their bacterial hosts. These findings provide a comprehensive understanding of cross‐kingdom microbial interactions and phage‐mediated gene exchange under dietary influences, opening new avenues for precise nutritional interventions targeting the gut microbiota.

## 5 Methods

### 5.1 Retrieval of Metagenomic Data Related to Dietary Intake

To construct a comprehensive lexicon of nutritional food components, we integrated data from multiple sources, including nutrient databases, food encyclopedias, and lists of edible items. Based on this lexicon, in conjunction with the keywords “diet,” “nutrition,” and “metagenome,” we formulated a composite search query. We performed a batch search of the NCBI BioProject database using Biopython v1.80 [83, 84] to retrieve project identifiers (BioProject IDs), titles, and descriptions. Subsequently, these were linked to the SRA database to obtain sample metadata, including sample size, total base count, and experimental descriptions.

To ensure data quality and relevance, we utilized XML parsing techniques (ElementTree module) [85] to select high‐quality metagenomic datasets, retaining only samples with a total base count of ≥500 MB, thereby systematically excluding 16S rRNA amplicon sequencing projects. Following manual curation, we excluded projects lacking clear grouping information, those unrelated to diet, and any remaining 16S rRNA amplicon studies. The accuracy of the sample grouping was rigorously verified against the original publications. During this process, we curated key metadata, including diet type, geography (continent and country), medication use, age, and disease state.

This process yielded a final dataset comprising 6932 metagenomic samples from 62 distinct BioProjects. All samples were categorized into two main groups based on their intake characteristics: “dietary types” and “dietary components.” The classification criteria were as follows: (1) studies involving a defined dietary strategy (e.g., Mediterranean diet) or labeled with the term “diet” were classified as dietary types; (2) studies involving a single nutrient, compound, or additive (e.g., fructose, polyphenols) were classified as dietary components (see Data S1).

Specifically, 6057 samples associated with 24 dietary types were subdivided into 11 categories: high‐fiber diets (e.g., Mediterranean diet, whole grain diet; 9 studies, *n* = 1,653), fat‐related interventions (16 studies, *n* = 459, predominantly in animal models), vegetarian studies (vegetarian versus omnivore lifestyles; 8 studies, *n* = 2,626), caloric restriction (3 studies, *n* = 295), and therapeutic enteral nutrition (e.g., exclusive enteral nutrition for Crohn's disease; 3 studies, *n* = 377), fermented foods (2 studies, *n* = 112), gluten intake (2 studies, *n* = 206), urbanization‐related dietary shifts (3 studies, *n* = 196), military ready‐to‐eat meals (MRE; 1 study, *n* = 65), low‐carbohydrate diets (1 study, *n* = 48), and protein interventions (1 study, *n* = 20).

Additionally, 3545 samples associated with 44 dietary components were classified into seven categories of specific components, including oligosaccharides (e.g., FOS, acarbose; 6 studies, *n* = 374), polysaccharides (e.g., resistant starch, inulin; 7 studies, *n* = 217), polyphenols (3 studies, *n* = 240), probiotics (7 studies, *n* = 270), different‐source protein (4 studies, *n* = 177), and other additives (e.g., sweeteners, cholesterol‐modulating agents; 4 studies, *n* = 104), as well as a large cohort examining alcohol consumption gradients (2 studies, *n* = 2,165). Notably, some samples that examined specific components within particular dietary patterns were included in both categories to support the analyses at different levels. The included studies employed a rigorous experimental design with parallel, washout, or baseline controls to ensure a high‐quality dataset for the meta‐analysis.

Raw sequencing data for all included metagenomic samples were downloaded from NCBI using the prefetch function of the SRA Toolkit v2.5.7 [85] and were subsequently converted to FASTQ format using the fasterq‐dump command for local storage and analysis.

### 5.2 High‐Fat Diet With Fucoidan Intervention in Animal Experiments

Animal procedures were approved by the Animal Welfare Ethics Committee of China Agricultural University (Approval Number: A2052020490‐4‐1) and complied with all relevant regulations. SPF‐grade female C57BL/6J mice (6 weeks old) were obtained from Beijing Huafukang Bioscience Co., Ltd., China (Laboratory Animal Production License: SCXK (Beijing) 2024‐0003). The mice were acclimatized for 7 days under identical pathogen‐free conditions (6 mice/cage, 12 h light:dark cycle) with ad libitum access to water and standard chow (D12450J, New Brunswick, NJ, USA). Following acclimation, mice were randomly assigned to three experimental groups (*n* = 6/group): control (CON) receiving standard chow D12450J; high‐fat diet (HFD) fed 60% high‐fat chow D12492 (New Brunswick, NJ, USA); fucoidan intervention (FUC) maintained on D12492 diet with daily oral gavage of 0.6 g/kg body weight fucoidan sulfate (purity > 90%, fucose content ≥ 20%, sulfate groups ≥ 25%; Qingdao Bright Moon Marine Fucoidan Biotechnology Co., Ltd., China) dissolved in sterile anaerobic phosphate‐buffered saline (PBS) from week 4 to week 7. The CON and HFD groups received equivalent volumes of PBS via gavage. Body weights were monitored weekly throughout the 7‐week study period. Fecal samples were collected from all groups on days 0, 10, 14, and 18 of the fucoidan intervention phase (weeks 4–7) and stored at −80°C for subsequent metagenomic analysis.

### 5.3 Shotgun Metagenome Sequencing

Total genomic DNA was extracted from stool (0.15 g) using a FastPure Stool DNA Isolation Kit (magnetic beads) (MJYH, Shanghai, China) according to the manufacturer's instructions. The concentration and purity of the extracted DNA were determined using SynergyHTX and NanoDrop2000, respectively. DNA quality was checked using a 1% agarose gel. The DNA extract was fragmented to an average size of approximately 350 bp using Covaris M220 (Gene Company Limited, China) for paired‐end library construction. A paired‐end library was constructed using NEXTFLEX Rapid DNA‐Seq (Bioo Scientific, Austin, TX, USA). Paired‐end sequencing was performed on Illumina NovaSeq X Plus (Illumina Inc., San Diego, CA, USA) at Majorbio Bio‐Pharm Technology Co., Ltd. (Shanghai, China) using the NovaSeq X Series 25B Reagent Kit according to the manufacturer's instructions (www.illumina.com).

### 5.4 Preprocessing of Metagenomic Data

Raw reads were processed using Fastp v0.23.3 [86] with the default settings to remove adapter sequences and low‐quality reads. Quality‐controlled reads were aligned to host reference genomes (Human: Ensembl GCF_000001405.39; Mouse: Ensembl GCF_000001635.27) using the BWA v0.7.17 [87] algorithm with default parameters, generating SAM files. SAMtools v1.6 [88] converted SAM to the BAM format, filtered unmapped non‐host reads (samtools view ‐bF 12) and coordinate‐sorted reads (samtools sort), and converted them to the FASTQ format (samtools fastq). Taxonomic classification based on reads of viruses and bacteria was conducted through Kraken2 v2.1.2 and Bracken v2.8 [89], retaining phage or bacterial species with relative abundance of >0.2 or detected in ≥1% of samples.

Target sequences were indexed using BWA v0.7.17 [87], and the sample reads were aligned using BWA‐MEM. Uniquely mapped reads were counted using SAMtools v1.6 (view ‐F 4) [88]. SeqKit v2.4.0 [90] was used to determine the target length (L) and total number of reads per sample (N). Normalized abundance was calculated as follows: Abundance = (mapped reads)/(L × N) × 10^10^.

### 5.5 Metagenomic Assembly and Phage Annotation

Quality‐controlled reads were filtered to remove Kraken2‐identified bacterial and eukaryotic sequences using SeqKit v2.4.0 [90]. Remaining reads were assembled into contigs using metaSPAdes v3.15.5 [91] (‐meta). Viral contigs were identified by VirSorter2 v2.2.4 [92] (‐min‐length 1000 ‐j 4 all) and quality‐assessed with CheckV v1.0.1 [93], removing contigs with “not‐determined” status. The BLAST v2.14.0+ [94, 95] reference library was based on the IMG/VR database [96] for viral species and bacterial host annotation using best‐hit alignments (>90% identity, E‐value < 1e‐5). Viral contigs were clustered into non‐redundant viral operational taxonomic units (vOTUs) at ≥85% identity using CD‐HIT v4.8.1 [97]. The phage lifestyle prediction for each viral contig was performed using PHACTS v1.8 [98] and BACPHLIP v1.0 [99].

To quantify phage lifestyle dynamics, we established a lytic–lysogenic index (LLI), defined as the ratio of virulent to temperate phage relative abundance, with the relative abundance determined by normalizing contig counts to total viral contigs per sample, to characterize the degree of change in phage lifestyle.

### 5.6 Phylogenetic Analysis of Diet‐Associated Gut Virome

Viral contigs assessed as “complete_genomes” by CheckV v1.0.1 were functionally annotated with VIBRANT v1.2.1 [100] (‐virome), retaining those encoding ≥2 essential structural genes (large terminases, portal proteins, and major capsid proteins). Open reading frames (ORFs) were predicted and translated using Prodigal v2.6.3 (‐p meta ‐g 11) [101] and then aligned to the NCBI non‐redundant protein (NR) database using DIAMOND v2.0.15 [102] (BLASTx mode, identity > 90%, E‐value < 1e‐5). Significant matches were stringently validated using BLASTp v2.14.0+ (identity > 90%, E‐value < 1e‐7). The taxonomic lineages (phylum to species) of NR entries were parsed using TaxonKit v0.8.0 [103]. Viral species were annotated based on the most frequent viral taxonomic unit (typically at the family/genus level), and bacterial hosts were assigned by selecting the highest‐frequency genus‐level taxon from the bacterial annotations, with crAssphage hosts manually assigned to *Bacteroides* following curation.

For phylogenetic reconstruction, protein sequences were clustered into orthologous groups with OrthoFinder v2.5.4 [104] (‐M msa), retaining clusters present in ≥30% of samples. After multiple sequence alignment (MAFFT v7.520 [105], –auto) and trimming of low‐conservation sites (TrimAl v1.4.1 [106], ‐gt 0.6), maximum‐likelihood trees were constructed using the GTR+CAT model in FastTree v2.1.11 [107] (‐gtr ‐gamma ‐nt) with 100 bootstrap replicates for node support.

### 5.7 Phage–Bacteria Correlation Analysis

Phage–bacteria associations were established through taxonomic nomenclature based on Kraken2‐annotated taxonomic lineages: a phage–bacterium pair was considered to have potential interaction with inferred infectivity if the first genus‐level taxonomic unit in the phage species name (prefix “s_”) exactly matched the bacterial genus name (e.g., “s__Bacteroides_phage” and “s__Bacteroides_plebeius”). The crAssphage was manually matched to Bacteroidetes. Spearman's correlation coefficient (*ρ*) was calculated to quantify the abundance correlation between each phage–bacterium pair (*P*_adj_ < 0.05 with Benjamini–Hochberg correction).

Phage–bacteria correlations were grouped by diet types, bacterial host genus, and intervention status (control vs. intervention) to calculate the median of absolute Spearman's *ρ* (|*ρ*|). Changes in interaction strength between the control and intervention groups were assessed using the Wilcoxon rank‐sum test and the comparison of median |ρ| values, with the direction of change defined as: “Increased” (|*ρ*|_intervention_ > |*ρ*|_control_, *p* < 0.05), “Decreased” (|*ρ*|_intervention_ < |*ρ*|_control_, *p* < 0.05), or “non‐significant” (*P* ≥ 0.05).

Bacterial taxa with significant changes in abundance (LDA score > 2, Kruskal–Wallis *p* < 0.05) following different dietary intakes were identified using LEfSe analysis, integrating these bacteria and defining them as diet‐associated bacteria (DABs). Phages associated with DABs were then selected, and for each, abundance change trends across all dietary intervention projects were determined; the number of projects where phage abundance increased after the intervention (N_increase_) and the number where it decreased (N_decrease_) were counted. If N_increase_ > N_decrease_, the overall trend of abundance for that phage was considered as “increasing” and vice versa. For each phage with a defined trend, the concordance of the abundance shifts with the bacterial host(s) was quantified. The number of projects where both the phage and its host changed in the same direction (i.e., both increased or both decreased; N_concordant_) and the number of projects where they changed in opposite directions (N_discordant_) were counted. The proportions of concordant changes (N_concordant_/(N_concordant_ + N_discordant_)) and discordant changes were calculated for each phage.

### 5.8 Identification of Horizontal Gene Transfer Events

Quality‐controlled reads were assembled into contigs using metaSPAdes v3.15.5 (‐meta) and binned into metagenome‐assembled genomes (MAGs) via MetaBAT2 v2.15 [108] (‐minContig 1500, ‐maxEdges 200). Taxonomic annotation of MAGs was performed by aligning them to the NCBI non‐redundant nucleotide (NT) database using BLASTn v2.14.0+ (identity > 90%, E‐value < 1e‐5). Horizontal gene transfer (HGT) events and associated transferred genes were identified at the phylum/class/order/family (pcofg) level with MetaCHIP v1.10.13 [109] (≥75% gene cluster coverage, ≥80% amino acid identity, ≥200 bp alignment length). The transferred genes identified were functionally annotated using eggNOG‐mapper v2.1.11 [110] and aligned against self‐assembled phage contigs using BLASTn v2.14.0+ (identity > 90%, E‐value < 1e‐5) to screen for phage‐mediated HGT events. To assess the dietary effects of HGT, genes encoding HGT‐associated enzymes (integrases, transposases, and recombinases) in phage contigs, annotated using VIBRANT v1.2.1 (‐virome), were extracted using SeqKit v2.4.0. The abundances of these genes were quantified across different dietary conditions and time series.

To explore the functional characteristics of the transferred genes during HGT events, we performed KEGG Orthology (KO) annotation on genes that showed significant changes in normalized abundance between day 0 and day 10 (Wilcoxon rank‐sum test, *p* < 0.05) across all HGT events.

### 5.9 Auxiliary Metabolic Genes Analysis

Auxiliary metabolic genes (AMGs) were identified from viral contigs using VIBRANT v1.2.1 (‐virome). To reconstruct a phylogeny of cross‐kingdom AMGs, ORFs for contigs containing bacterial sequences were predicted with Prodigal v2.6.3 (‐f gff ‐p meta), generating nucleotide sequences (.fna). These gene sequences were aligned against VIBRANT‐preannotated viral AMGs using BLASTn v2.14.0 (‐outfmt 6), filtering putative homologous genes (E‐value < 1e‐5, bitscore > 90). Non‐redundant AMGs of bacterial origin were extracted by retaining the first unique record per query ID after deduplication. The bacterial AMG set was integrated with the VIBRANT‐derived viral AMGs to construct a cross‐kingdom dataset. Multiple sequence alignments were performed using MAFFT v7.520 (‐auto), followed by maximum‐likelihood phylogenetic reconstruction using the ModelFinder algorithm in IQ‐TREE v1.6.12 [111] (‐m MF ‐redo).

To identify AMGs involved in amino acid metabolism that were altered by HFD and subsequently reversed by FUC, all AMGs were mapped to KO identifiers and filtered for those associated with amino acid metabolism pathways. KOs showing significant changes in normalized abundance between day 0 and day 10 in both the HFD and FUC groups (Wilcoxon test, *p* < 0.05) were retained. From these, we selected KOs that met two criteria: (1) expression in the HFD group was elevated compared to its own day‑0 baseline (positive percent change), and (2) at the same time point (day 10, 14, or 18), expression in the FUC group was lower than that in the HFD group. This two‐step filtering pinpointed KOs that were upregulated by HFD and downregulated by FUC. Selected KOs were grouped by annotated metabolic pathway (e.g., cysteine and methionine metabolism, lysine degradation) and, within each pathway, sorted by the magnitude of HFD‐induced up‐regulation at day 10.

### 5.10 Statistical Analysis

To minimize cross‐study heterogeneity, we applied a consistent bioinformatics pipeline across all samples, utilized ConQuR v1.3.3 [112] for batch effect correction, and prioritized intra‐project comparisons between the intervention and control groups. PERMANOVA (vegan v2.6.4::adonis2; Bray–Curtis distance, 999 permutations) was used to quantify the variance (*R* ^2^) explained by multifactorial drivers (diet type, dietary components, geography, age, and disease status); age and disease status were included as covariates in the multivariate models to control for confounding factors (Data S3). β‐Diversity was evaluated by balancing sample sizes through random subsampling. The median Bray–Curtis (BC) distance between the pre‐ and post‐intervention samples was calculated (vegan v2.6.4::vegdist) and assessed using Wilcoxon rank‐sum tests. This subsampling procedure was repeated 100 times, and the median BC distance and *p*‐value for each iteration were recorded. Differentially abundant taxa were identified using LEfSe analysis (LDA > 2, Kruskal–Wallis *p* < 0.05). Bacterial community dissimilarities in the animal experiments were measured using non‐metric multidimensional scaling (NMDS) based on BC distances, with ordination quality assessed using stress values. Group separation was tested using Adonis (*p* < 0.001, 999 permutations), and independent NMDS plots were generated for days 0, 10, 14, and 18 to visualize temporal dynamics.

For hypothesis testing, the differences between the two groups were assessed using two‐sided Wilcoxon rank‐sum tests. For comparisons of three or more groups, parametric data (e.g., body weight) were analyzed using one‐way ANOVA with Tukey's post‐hoc test, whereas non‐parametric data (e.g., LLI changes) were analyzed using the Kruskal–Wallis test with Dunn's post‐hoc test. All tests were two‐sided with a significance level (α) of 0.05, and *p*‐values were adjusted for multiple comparisons using the Benjamini–Hochberg method. Significance is indicated as follows: *p < 0.05, **p < 0.01, ***p < 0.001, ****p < 0.0001; comparisons without asterisks are not significant. Different letters in bar charts denote statistically significant differences (*p* < 0.05). Box plot elements are defined as follows: center line, median; box limits, upper and lower quartiles; whiskers, 1.5× interquartile range; points, outliers.

All statistical analyses were performed in RStudio using R (v4.3.1), with the vegan package (v2.6.4) for community ecology analyses and the ggplot2 package (v3.5.1) for visualization.

## Author Contributions

F.Z. collected and analyzed metagenomic raw data. R.Z. drafted the initial iteration of the manuscript and figures. R.W. conducted animal experiments, sample collection, and testing. H.F. managed and maintained metagenomic data. Y.H. guided the animal experiments. W.S. participated in data analysis and statistics. J.W. conceived and supervised the study and revised the paper. All authors reviewed subsequent iterations and approved the final version for submission.

## Funding

This study was supported by the National Key Research and Development Program of China (2022YFA1304102), the National Natural Science Foundation of China (T2341010 and 32370053), and the 2115 Talent Development Program of China Agricultural University.

## Conflicts of Interest

The authors declare no conflicts of interest.

## Supporting information




**Supporting File 1**: advs74765‐sup‐0001‐SuppMat.pdf

**Supporting File 2**: advs74765‐sup‐0002‐Data.zip

## Data Availability

The summary of diet‐associated metagenomes is available in Supplementary Data S1. The metagenomic sequencing data of animal experiments in this study have been deposited in the NGDC GSA database (Accession Number: PRJCA044097). The data analysis and plotting code in this study is available at https://github.com/zhangruiqi32/diet_phage_bacteria.
