# Supplementary material for: Alternating High‐Fat and Polysaccharide Diets Modulates Gut Phage‐Bacterial Interplay
Source: Adv Sci (Weinh). 2026 Mar 12;13(29):e16916. doi: 10.1002/advs.202516916 (PMC13205691; doi:10.1002/advs.202516916)
Supplement: Supplementary file 1 — Supporting File 1: advs74765‐sup‐0001‐SuppMat.pdf [file ADVS-13-e16916-s001.pdf]

Supplementary Information

ALTERNATING HIGH-FAT AND POLYSACCHARIDE DIETS MODULATES GUT PHAGE–BACTERIAL INTERPLAY

Fengxiang Zhao[#], Ruiqi Zhang[#], Rujun Wei, Huimin Fan, Yongfei Hu, Wenyu Shi, Jinfeng Wang[*]

*To whom correspondence should be addressed.

E-mail: Jinfeng Wang (E-mail: wangjf@cau.edu.cn)

This PDF file includes:

Supplementary Figures 1 to 10

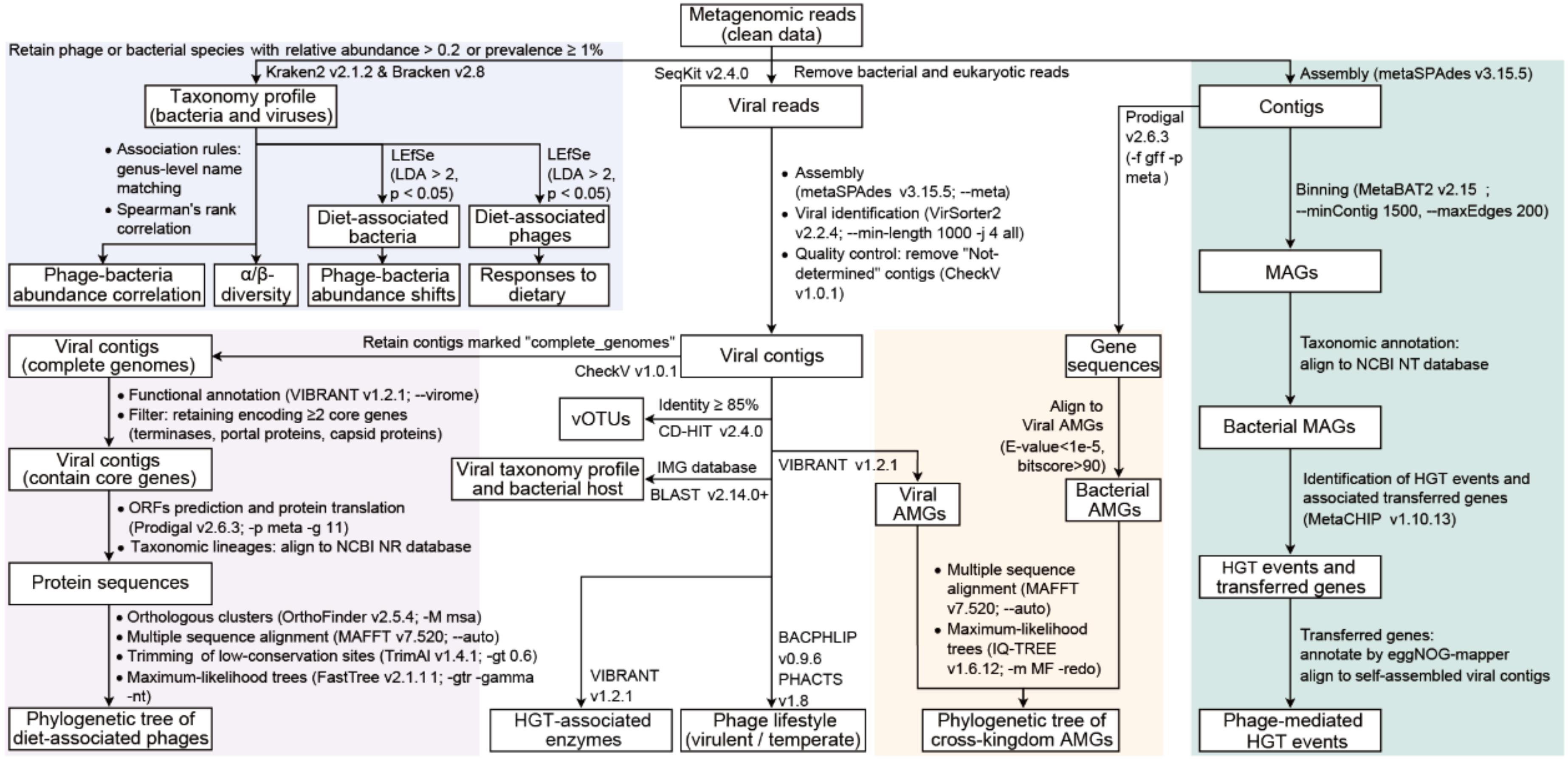

Supplementary Fig. 1 Analytical workflow for metagenomic data.

The analytical workflow for metagenomic data following quality control.

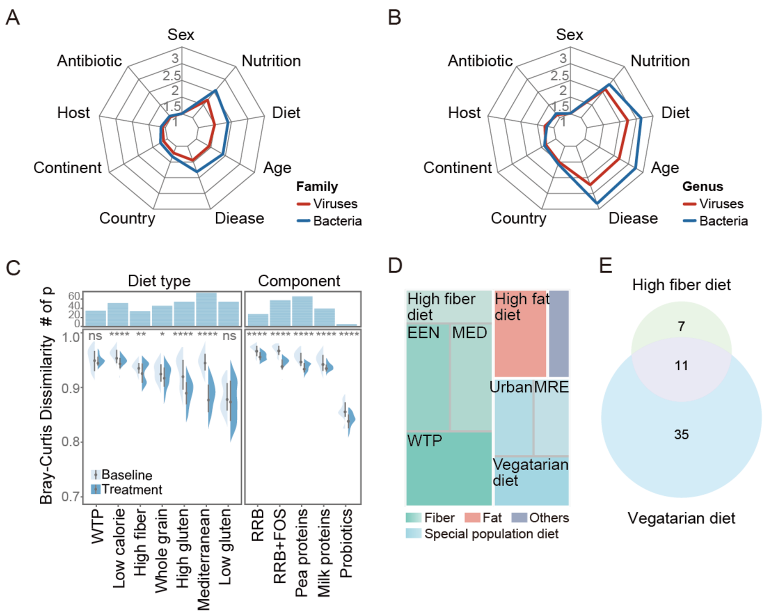

Supplementary Fig. 2 Dietary modulation of gut bacteriome and virome.

A-B. The effect size of diet and other factors to shape the gut virome and bacterial communities. This radar chart compares the effect size (PERMANOVA R^2, Bray-Curtis (BC) dissimilarity, 999 permutations) of various factors on gut virome (red line) and bacteriome (blue line) at family (A) and genus (B) levels. C. Same diet increases inter-individual similarity of the gut virome. The lower panel shows BC distances of virome between treatment and baseline in 100 samplings (n = 18 per diet type per sampling); violin plots indicate the median, interquartile range, and the density distribution of BC distances. The upper panel displays the number of significant differences in BC distance before and after dietary intake (two-sided Wilcoxon rank-sum test). D. Proportion of dietary types significantly altering phage abundance (LEfSe, LDA score > 2, Kruskal–Wallis P < 0.05). Colors represent different dietary types. E. Shared diet-responsive phages between vegetarian and high-fiber diets. The circle areas are proportional to the number of phage species showing significant abundance changes (LEfSe, LDA score > 2, Kruskal–Wallis P < 0.05) in response to each diet. Statistical significance was determined by the Wilcoxon test: *P < 0.05, **P < 0.01, ***P < 0.001, ****P < 0.0001; comparisons without asterisks are not significant. Abbreviations: WTP, Whole-grains, Traditional Chinese medicinal foods and Prebiotics diet; RRB, (poly)phenol-dense red raspberries; FOS, fructo-oligosaccharide; MED, Mediterranean diet; EEN, Exclusive enteral nutrition; MRE, Ready-to-eat Meal; Urban, High urbanization diet.

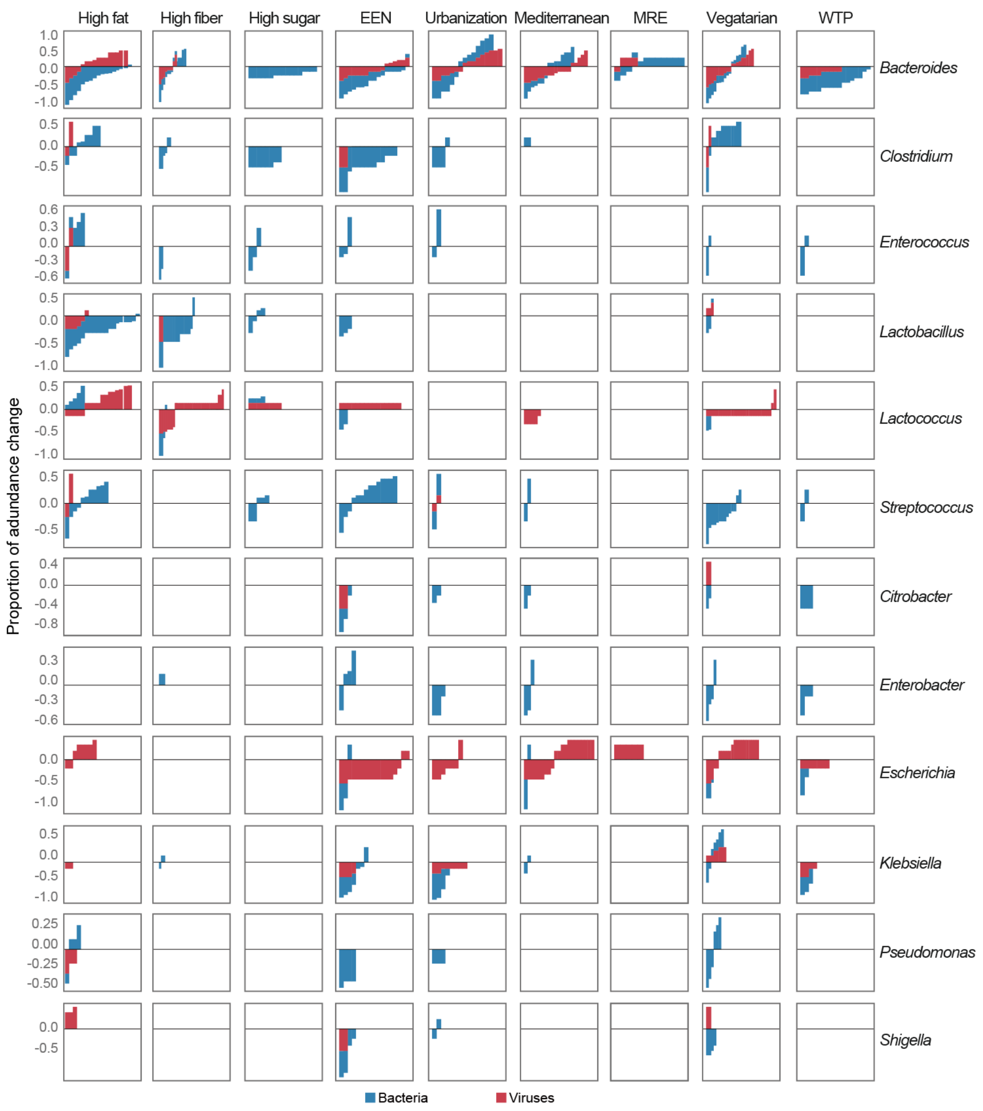

Supplementary Fig. 3 Diet-induced abundance changes in phages and their bacterial hosts.

The direction of abundance changes for phages (red bars) and their corresponding bacterial hosts (blue bars) in response to various dietary interventions. Each row represents a bacterial host genus. For each diet and host combination, the bars on the positive and negative y-axes indicate the proportion of phage/bacterial species within that genus that increased and decreased in abundance, respectively, relative to the total number of species identified for that genus. EEN, Exclusive enteral nutrition; MRE, Ready-to-eat Meal; WTP, Whole-grains, Traditional Chinese medicinal foods and Prebiotics diet.

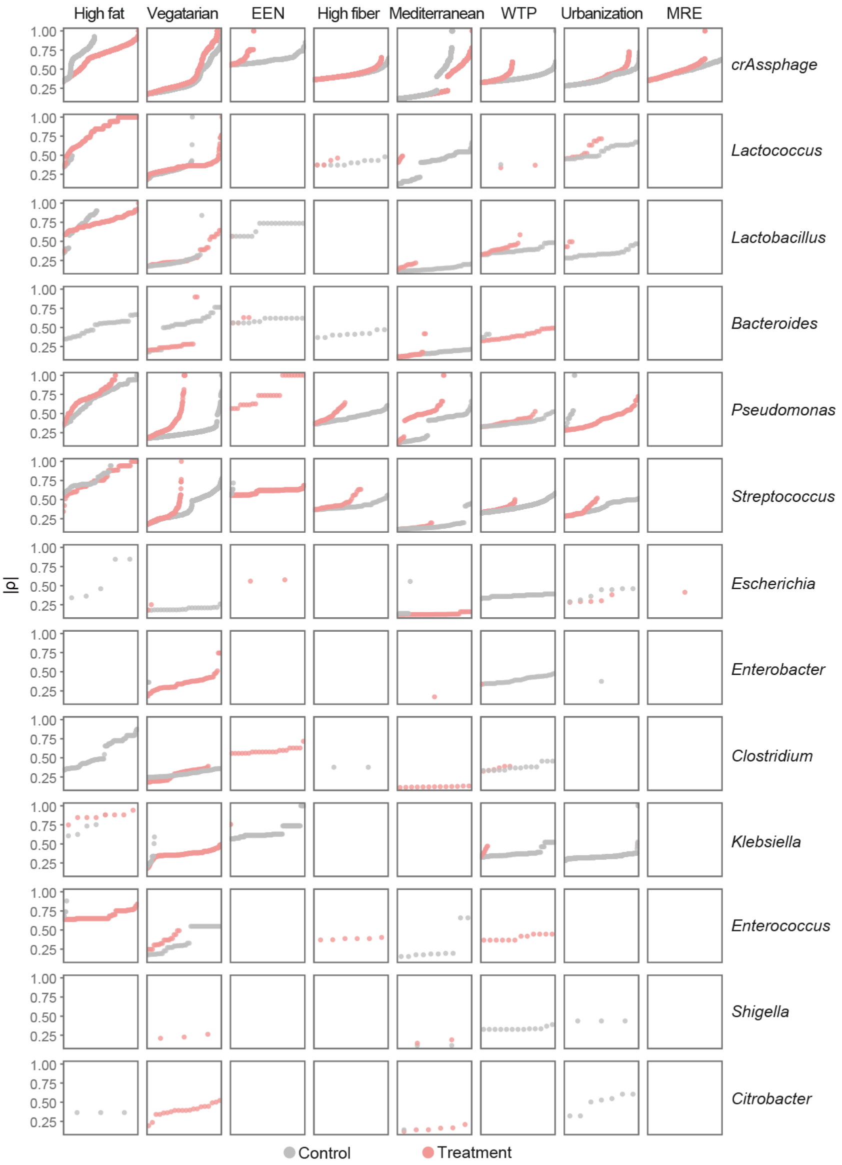

Supplementary Fig. 4 Diet-driven shifts in phage–bacteria interactions.

The strength (absolute Spearman's correlation coefficient, |ρ|) of significant ($P_{adj} < 0.05$ with Benjamini–Hochberg correction) correlations for phage–bacteria pairs. Data are stratified by bacterial host genus (rows) and dietary types (columns). Points from dietary treatment groups are colored red, while those from corresponding control groups are grey. EEN, Exclusive enteral nutrition; MRE, Ready-to-eat Meal; WTP, Whole-grains, Traditional Chinese medicinal foods and Prebiotics diet.

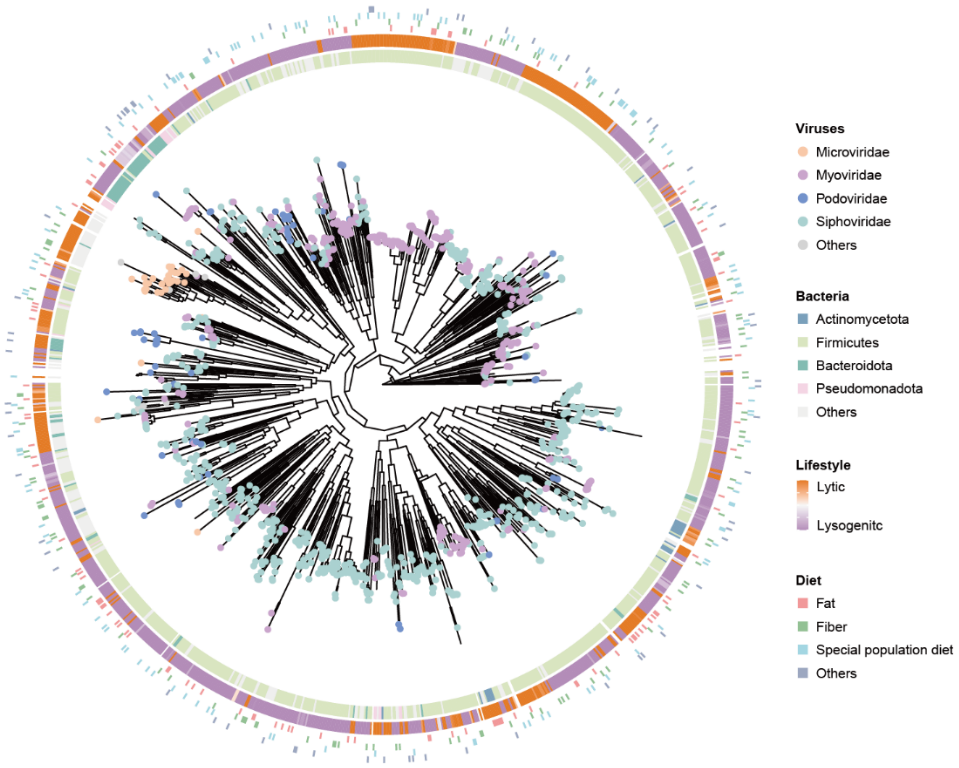

Supplementary Fig. 5 Phylogenetic relationships of diet-associated gut viromes. A maximum-likelihood phylogenetic tree was constructed from high-quality, complete viral genomes (as assessed by CheckV v1.0.1, encoding ≥ 2 essential structural genes; see Methods). Each point represents an individual phage, colored by its taxonomic family. Concentric rings, from inner to outer, display annotations for each phage: (1) the predicted bacterial host, colored by phylum; (2) the predicted lifestyle; and (3) associated dietary context.

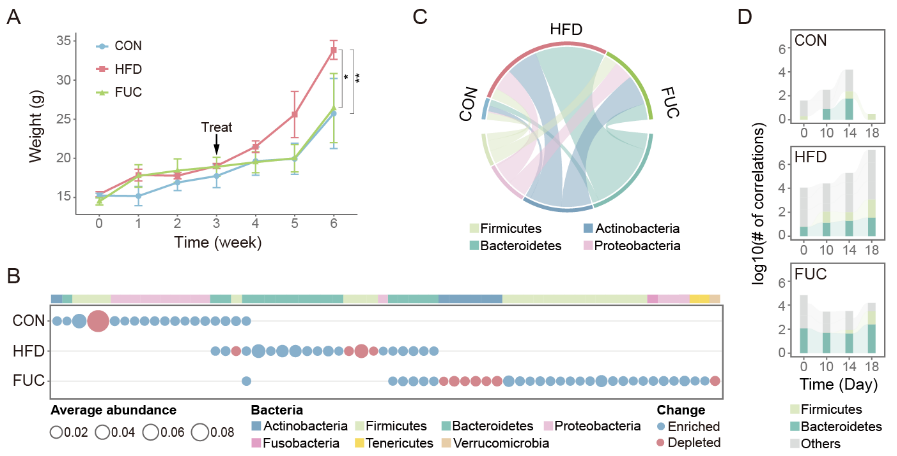

Supplementary Fig. 6 Fucoidan mitigates HFD-induced weight gain and remodels the gut microbiota.

A. FUC treatment attenuates HFD-induced body weight gain. This line graph tracks the body weight of mice in each group over time. At the 6-week endpoint, mice on the HFD were significantly heavier than both CON and FUC mice, while the FUC and CON groups were not significantly different (one-way ANOVA with Tukey's test, $P<0.05$). Data are mean ± s.d. ($n = 6$). B. Dietary interventions selectively enrich or deplete specific bacterial genera. Dot plot of differentially abundant bacterial genera at day 10 of the intervention, as identified by LEfSe analysis (LDA score > 2, Kruskal–Wallis $P < 0.05$). Point size is proportional to the genus's average relative abundance. Colors indicate whether the genus was enriched (blue) or depleted (red) in the treatment group compared to the control. The top color bar indicates the phylum of each genus. C. The profile of bacteria-associated phages is reshaped by diet. This chord diagram shows the taxonomic composition (at the host-phylum level) of phages that significantly correlate (Spearman's ρ, $P_{adj} < 0.05$ with Benjamini–Hochberg correction) with all bacteria at day 10 of the intervention, (at the host-phylum level). D. Temporal dynamics of phage–bacterial correlations across dietary intervention groups. The stacked bar plots with alluvial flows illustrate the log10-transformed number of significant correlation pairs between phages and their bacterial hosts (Spearman's ρ, $P_{adj} < 0.05$ with Benjamini–Hochberg correction) over time (Day 0, 10, 14, and 18) in CON, HFD, and FUC groups. Colors represent the bacterial host phylum, with alluvial flows depicting the shifts in correlation patterns across time points within each dietary group. CON, control diet; HFD, high-fat diet; FUC, HFD with fucoidan supplementation. *$P < 0.05$, **$P < 0.01$.

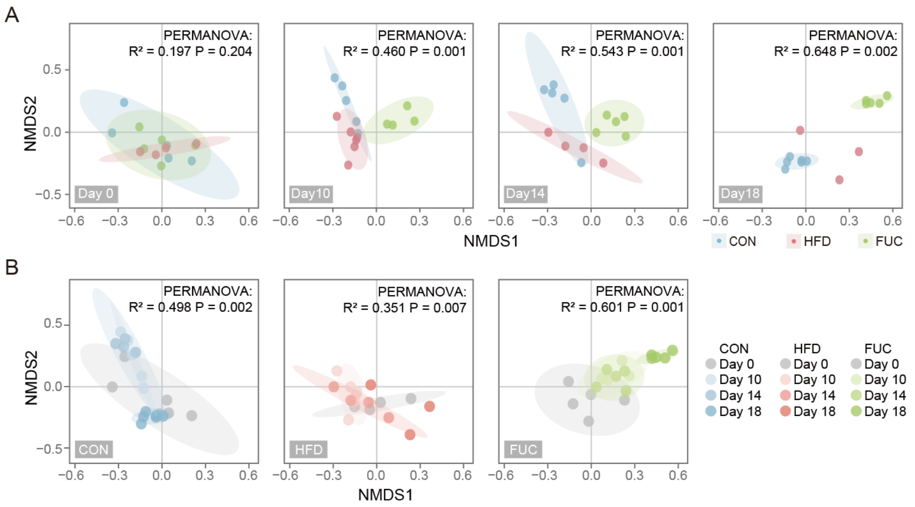

Supplementary Fig. 7 Temporal dynamics of gut microbiota structure.

A. Time-point specific non-metric multidimensional scaling (NMDS) analysis of gut bacterial communities. NMDS plots based on Bray-Curtis dissimilarities are shown separately for days 0, 10, 14, and 18 (global NMDS stress = 0.16). Points represent individual samples colored by dietary group (CON: blue, HFD: red, FUC: green). Ellipses represent 90% confidence intervals. Statistical significance of group separation at each time point was assessed using PERMANOVA (999 permutations), with R^2 and P-values displayed in each panel. B. Longitudinal trajectories of gut microbiota within dietary groups. NMDS plots faceted by diet (CON, HFD, FUC) visualize the temporal shift of bacterial communities from Day 0 to Day 18 (global NMDS stress = 0.16). Points are colored by a gradient representing time points within each diet group (Day 0: grey; Day 10, 14, 18: intensifying shades of blue, red, and green for CON, HFD, and FUC, respectively). PERMANOVA results assessing the effect of time within each diet group are shown. CON, control diet; HFD, high-fat diet; FUC, HFD with fucoidan supplementation.

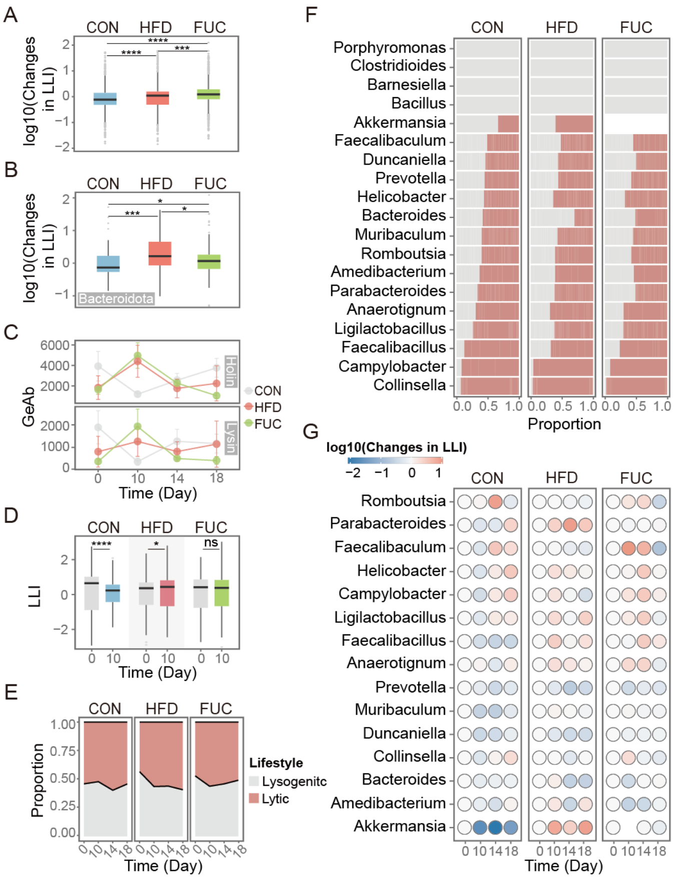

Supplementary Fig. 8 HFD and FUC drive the transformation of phage lifestyles. A-B. Diet-driven shifts in phage lifestyle balance. These box plots represent the log10-transformed fold change (Day 10 vs. Day 0) in the lytic-lysogenic index (LLI) across the three dietary groups, calculated as $\log_{10}$(LLI_day10/LLI_day0 + 1), where LLI = | $\log_2$(virulent vOTU abundance+0.1 / temperate vOTU abundance+0.1) |. The analysis is shown for phages infecting DABs (A) and Bacteroidetes (B) specifically. C. Temporal dynamics in the normalized abundance (GeAb) of lysis-related genes (holins and lysins) in phages across dietary intervention groups (CON, HFD, and FUC). Data are presented as mean ± s.e.m. Sample size n = 6 mice per group. D. Dietary intervention alters the LLI of DAB-associated phages. These box plots display the LLI for phages infecting DABs at baseline (Day 0) and after 10 days of intervention. E. Temporal dynamics of lifestyles for phages infecting DABs. F. Lifestyle composition of different phages at day 10 of the intervention. This bar chart shows the relative proportions of virulent (red) and temperate (grey) phage abundance within distinct phages grouped by host genus. G. Temporal dynamics of lifestyle changes in phages of DABs. The color of each dot represents the log10-transformed fold change in the LLI for phages of DABs (grouped by host genus) at a given time point (Day 10, 14, or 18)

relative to baseline (Day 0). LLI was calculated as the median of (virulent phage abundance + 1) / (temperate phage abundance + 1) across all phages infecting that host. Red points signify a shift toward lytic dominance, while blue points signify a shift toward lysogenic dominance. LLI is defined as the abundance ratio of virulent to temperate phages. Box plot elements are: center line, median; box limits, upper and lower quartiles; whiskers, 1.5 × interquartile range; points, outliers. Statistical significance was determined by the two-sided Wilcoxon rank-sum test: *$P < 0.05$, **$P < 0.01$, ***$P < 0.001$, ****$P < 0.0001$; comparisons without asterisks are not significant. CON, control diet; HFD, high-fat diet; FUC, HFD with fucoidan supplementation.

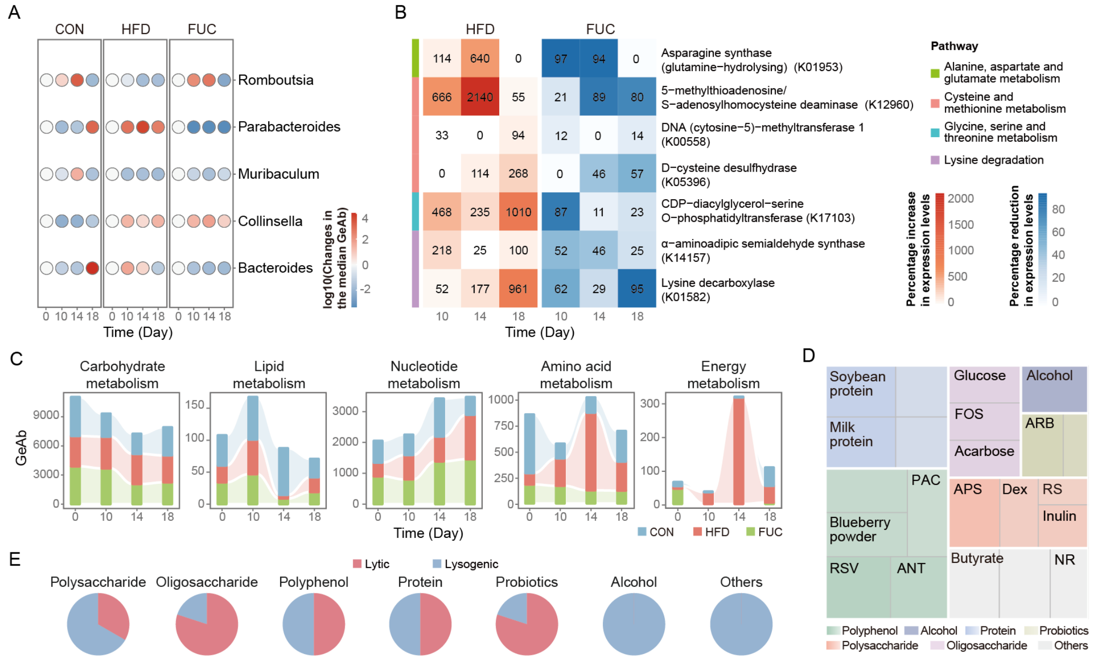

Supplementary Fig. 9 Temporal and component-specific responses of the gut virome to diet.

A. Temporal dynamics of HGT-related gene abundance in phages of DABs. The color of each dot represents the log10-transformed fold change in the median normalized abundance (GeAb) of HGT-related genes for phages of DABs at a given time point (Day 10, 14, or 18) relative to baseline (Day 0). Red points signify an increase in abundance, while blue points signify a decrease. B. The HFD-induced upregulation of amino acid metabolism-related AMGs and their reversal by FUC supplementation. The left panel shows the percentage increase in expression levels of specific AMGs in the HFD group relative to Day 0 baseline (red gradient) at different time points (Day 10, 14, or 18), while the right panel shows the percentage reduction in the FUC group relative to the HFD group at corresponding time points (blue gradient). Rows represent individual AMGs (annotated with KO IDs and gene names) grouped by metabolic pathway, and numbers within cells indicate the percentage change values. C. Temporal dynamics for AMGs abundance. These plots show the normalized abundance (GeAb) of AMGs from the entire virome (left 3) and phages infecting DABs (right 2) over the course of the different dietary intervention. D. Proportion of dietary components significantly altering phage abundance. E. Proportion of phage lifestyle after different dietary component intake. CON, control diet; HFD, high-fat diet; FUC, HFD with fucoidan supplementation. ANT, anthocyanidin; PAC, proanthocyanidin; RS, Resistant starch; APS, astraglus polysaccharide; NR, nicotinamide riboside; FOS,

fructooligosaccharide; RSV, resveratrol; Dex, dextrin; ARB, antibiotic resistant bacteria.

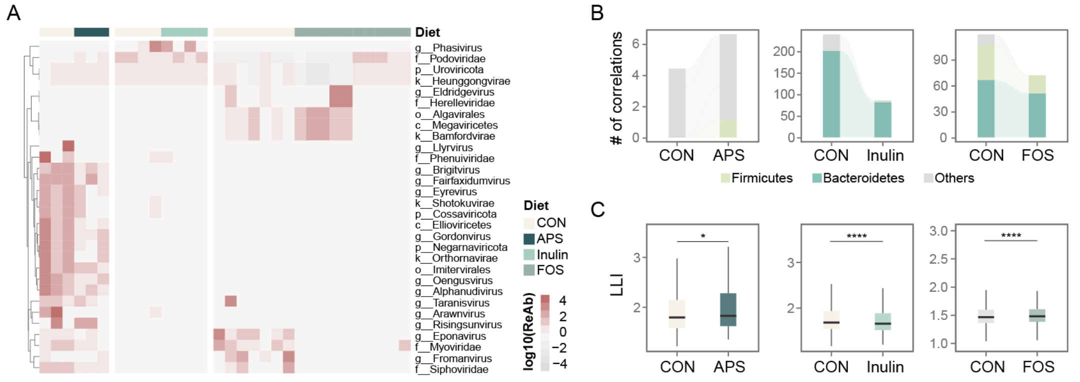

Supplementary Fig. 10 Broad regulatory effects of different saccharides on HFD-associated phages.

A. Abundance profiles of HFD-enriched phage taxa across different saccharide interventions under HFD conditions. Phage taxa significantly enriched under HFD conditions (identified by LEfSe analysis in ≥ 3 projects) are displayed in rows, with samples arranged by dietary intervention in columns. Color intensity indicates log10-transformed relative abundance (ReAb) levels. B. Modulation of phage–bacterial correlation networks by different saccharide interventions under HFD conditions. The stacked bar plots with alluvial flows illustrate the total number of significant correlations between phages and their bacterial hosts (Spearman's ρ, $P_{adj} < 0.05$ with Benjamini–Hochberg correction) across different dietary groups. Colors represent the bacterial host phylum, highlighting shifts in interaction patterns induced by different saccharides. C. Effects of different saccharide interventions on phage lifestyle balance under HFD conditions. These box plots display the Lytic-Lysogenic Index (LLI) across different dietary groups, calculated as LLI = $|\log_{10}$(virulent vOTU abundance+0.1 / temperate vOTU abundance+0.1)$|$. Box plot elements are defined as: center line, median; box limits, upper and lower quartiles; whiskers, 1.5 × interquartile range. Statistical significance between the intervention and control groups within each project was determined by the two-sided Wilcoxon rank-sum test with Benjamini–Hochberg adjustment: *$P < 0.05$, ****$P < 0.0001$; comparisons without asterisks are not significant. CON, control diet (HFD alone); APS, astraglus polysaccharide; FOS, fructooligosaccharide.
