# Supplementary material for: Alternating High‐Fat and Polysaccharide Diets Modulates Gut Phage‐Bacterial Interplay
Source: Adv Sci (Weinh). 2026 Mar 12;13(29):e16916. doi: 10.1002/advs.202516916 (PMC13205691; doi:10.1002/advs.202516916)
Supplement: Supplementary file 2 — Supporting File 2: advs74765‐sup‐0002‐Data.zip [file ADVS-13-e16916-s002.zip › advs74765-sup-0002-Data/Description of Additional Supplementary Files.docx]

**Description of Additional Supplementary Files**

**File Name:** Supplementary Data 1

**Description:** Metadata of 6,932 gut metagenomic samples, including host demographics, geography, health status, and dietary records.

**File Name:** Supplementary Data 2

**Description:** Category of standardizing raw diet types and components into harmonized categories.

**File Name:** Supplementary Data 3

**Description:** Supporting data for PERMANOVA (covariate-adjusted) of diet effects on virome/bacteriome and diet‑responsive phage taxa across dietary type.

**File Name:** Supplementary Data 4

**Description:** Supporting data for diet-associated bacteria taxonomy and mice body weights under different diets.

**File Name:** Supplementary Data 5

**Description:** Supporting data for high-fat diet (HFD) and fucoidan-induced phage lifestyle transitions.

**File Name:** Supplementary Data 6

**Description:** Supporting data for horizontal gene transfer (HGT) events and associated HGT gene annotations.

**File Name:** Supplementary Data 7

**Description:** Supporting data for auxiliary metabolic gene (AMG) metabolic distributions (lysogenic state), phage lifestyle associations in amino acid metabolism, and AMG-host associations across diets and timepoints.

**File Name:** Supplementary Data 8

**Description:** Supporting data for phage-bacteria interaction networks across dietary components.
